# ROBOTONT – Open-source and ROS-supported omnidirectional mobile robot for education and research

**DOI:** 10.1016/j.ohx.2023.e00436

**Published:** 2023-06-01

**Authors:** Renno Raudmäe, Sandra Schumann, Veiko Vunder, Maarika Oidekivi, Madis Kaspar Nigol, Robert Valner, Houman Masnavi, Arun Kumar Singh, Alvo Aabloo, Karl Kruusamäe

**Affiliations:** University of Tartu, Estonia

**Keywords:** Open-source, Educational robotics, ROS (Robot Operating System), Holonomic, Mobile robot, Software, Hardware, Electronics, Professional education, University teaching

## Abstract

In order to achieve visionary concepts such as Society 5.0 and Industry 5.0, there is a growing need for people who are able to create innovative robotic technologies. Training students to become such skilled professionals requires transitioning from often toy-like educational platforms with significant hardware limitations to costly research robots with full ROS (Robot Operating System) support. To aid in this transition, we propose Robotont – an open-source omnidirectional mobile robot platform with both physical hardware and a digital twin. Robotont enables robotics education with professional tools as well as provides researchers with a capable mobility platform for validating and demonstrating scientific results. Robotont has successfully been used for university teaching, professional education, and online courses about ROS and robotics.

## Graphical abstract

**Figure:**
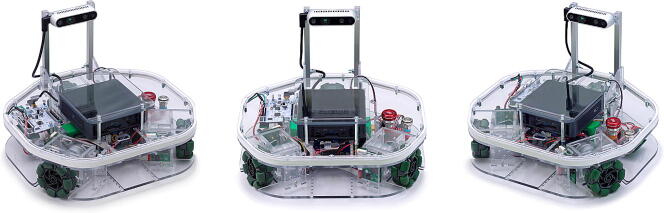

Specifications table.

**Table:** 

Hardware name	*Robotont, omnidirectional mobile robot*
Subject area	● Educational tools and open-source alternatives to existing infrastructure
Hardware type	● Robotics engineering ● Electrical engineering and computer science Mechanical engineering
Open-source license	*CERN-OHL-P-2.0 for the hardware* *Apache 2.0 for the software*
Cost of hardware	*2000 €*
Source file repository	https://doi.org/10.5281/zenodo.7897546 https://github.com/robotont/robotont-hardware-x-2023-replication-package https://github.com/robotont

## Hardware in context

In order to achieve visionary concepts such as Society 5.0 and Industry 5.0 [1], there is an increasing demand for people who are able to lead and support technological innovation, e.g. roboticists in the domain of engineering and manufacturing [2]. Obtaining a sufficient amount of experts for this purpose via skilling, reskilling, and upskilling requires different educational robotic platforms that enable hands-on experience (incl programming, deploying, and using) for a wide range of technological concepts such as manufacturing automation, shop floor logistics, human-robot collaboration, and fleet management. At the same time, in order to reach a sufficiently wide audience, educational platforms need to strike a balance between cost and technical performance to be affordable for educational institutions while facilitating the learning of advanced concepts.

More specifically, there is a demand for robotic system operators and developers who are proficient in ROS (Robot Operating System), which has become the de facto software framework for developing novel integrated robot solutions [3], [4]. A ROS-supported educational robot enables the learner, in addition to obtaining practical framework-specific skills, to become familiar with high-level robotics concepts (e.g. state estimation, mapping, kinematic solvers, motion planning, and object tracking), which can be considered relatively straightforward to deploy in ROS.

A majority of the existing educational platforms (e.g, OpenBot [5], DuckieTown [6], Mona [7], LEGO Mindstorms [8], mBot [8], e-puck [9], and GoPiGo3 [10]), aimed at beginners taking their first steps in the field, seek to maximize technical reliability and minimize the cost (<500 €). While such solutions are excellent for developing computational thinking in youth within the wide range of STEAM programmes, the limited hardware (e.g., reduced compute resources, minimalistic sensor kits, and modest physical features) of these robot platforms may fall short for professional training or higher education.

Often the next best choice after exhausting the capabilities of previously mentioned education robots is the group of research robots (e.g., ClearPath Jackal, PAL Robotics TIAGo, Mobile Industrial Robot MiR100, and Boston Dynamics Spot), with tentative unit prices ranging from 15,000 to 100,000 €. These robots offer reliable hardware capable of high-performance computational tasks as well as handling realistic physical loads. These robots generally have ROS support available and often these same robots are deployed with the end user, thus making knowledge transfer from the training setting to the application domain extremely straightforward. However, since these robots are not primarily intended for learning purposes, the high cost can limit the educators' options for offering training.

There is a clear need for robots that would enable learning professional tools without oversimplifying the associated concepts while being accessible to a wide audience of learners. Those goals are aided by open-source hardware and software that helps to bring down costs and support quick customization [11], [12]. In addition to providing an accessible platform for learners, open-source hardware in robotics paves the way to more standardized deployment and benchmarking of research outcomes [13], [14], [15].

In this article, we present Robotont – an open-source omnidirectional mobile robot for the primary purpose of enabling robotics education with professional tools. In terms of material cost, the robot hits the sweet spot between the first-stage educational robots and advanced research robots by aiming to meet the technical requirements of both domains. Therefore, Robotont is equipped with a powerful onboard computer, a depth camera, and three DC motors that are mounted at 120 degrees for versatile holonomic kinematics (i.e. the Kiwi drive). The entire software system is fully compatible with ROS and due to the advanced nature of hardware configuration, the platform can be used for learning high-level concepts enabled by ROS as well as a demonstrator for research outcomes in, e.g., transportation and swarm robotics.

## Hardware description

### Introduction

**Fig. 1:**
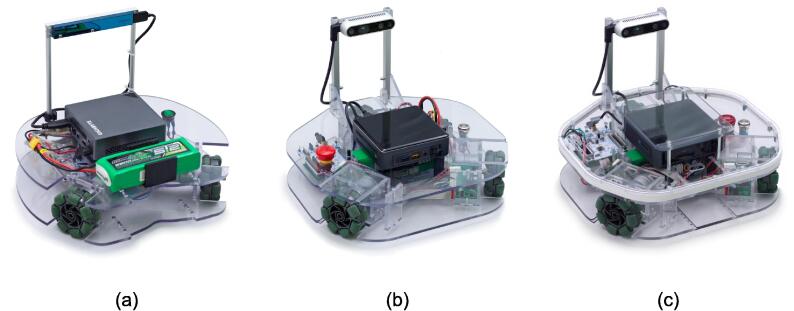
The evolution of the Robotont platform. (a) Generation 1, (b) generation 2, and (c) generation 2.1.

Robotont is an open-source hardware and software platform for advanced robotics education and research. It is an omnidirectional mobile robot with ROS support. To date, three versions of Robotont robots have been designed (Fig. 1) and this article delivers a comprehensive overview of the latest iteration of the system (Fig. 1-c). Even though the robot platform is designed to be reconfigurable allowing users and developers to add and replace sensors, computational units, etc., here we describe our standard configuration, which in addition to main mobility platforms consists of a system-on-module architecture PC and a USB-connected depth camera. To describe the robot in full, we provide individual subsections about its mechanical assembly (section “Mechanical assembly’), electronics (section “Electronics”), and software (section “Software”).

### Mechanical assembly

**Fig. 2:**
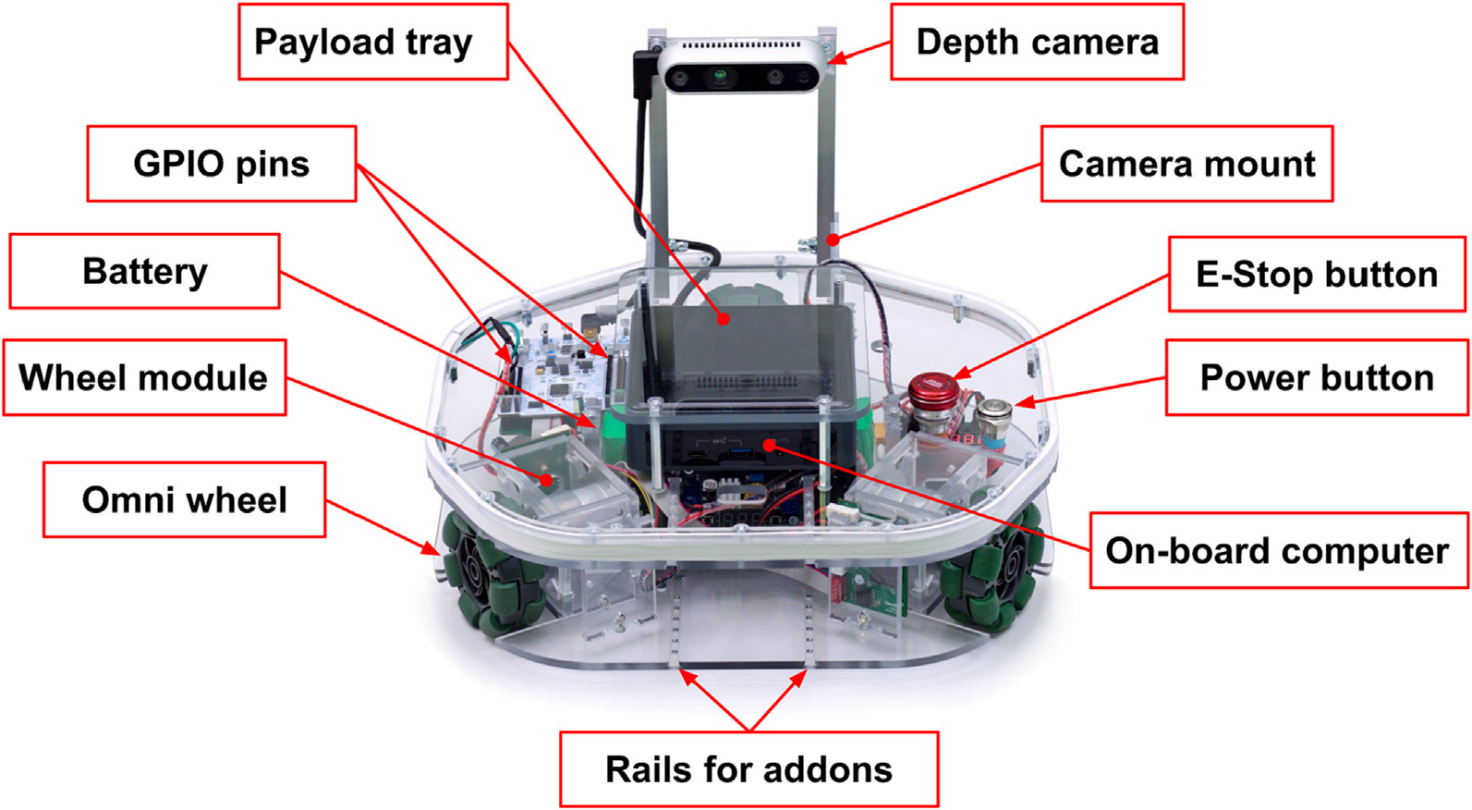
Fully assembled Robotont mobile robot.

The general mechanical assembly of the Robotont robot is depicted in Fig. 2. The polycarbonate main chassis houses the majority of the robot components: three wheel modules, electronic circuit boards for the main microcontroller, power management and motor control, as well as the power supply and wiring.

**Fig. 3:**
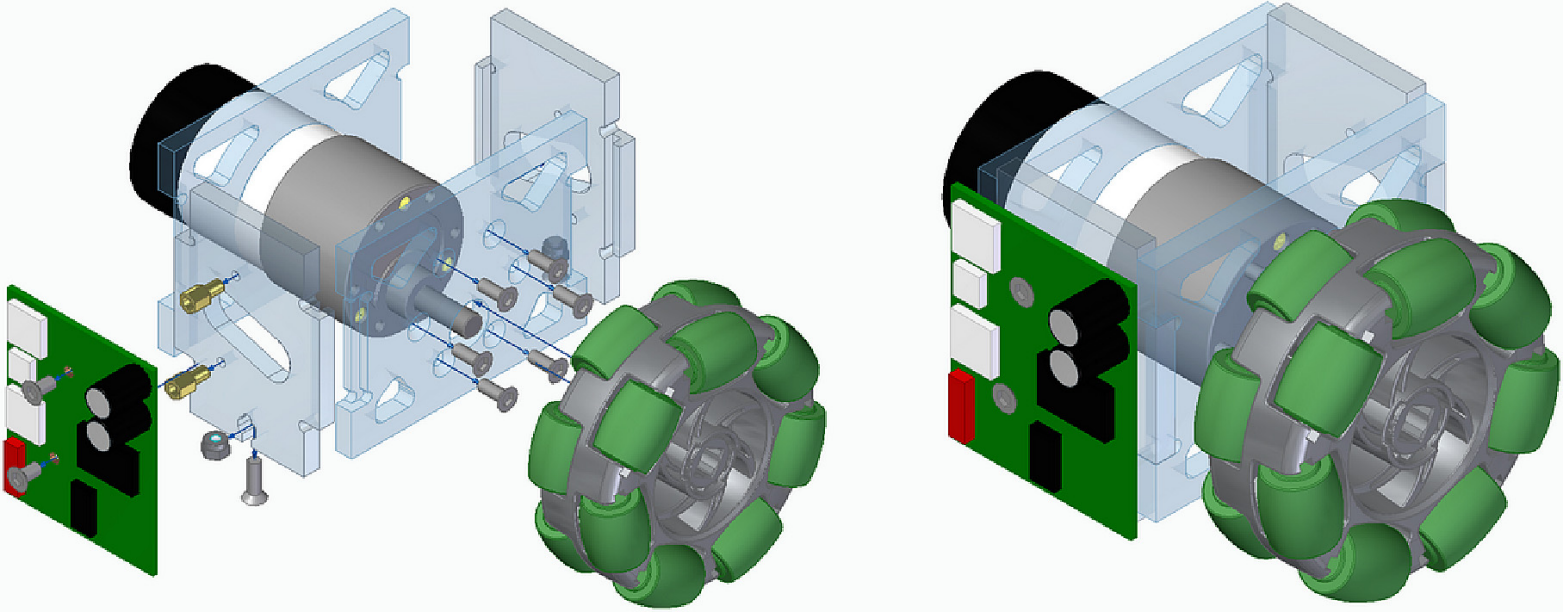
The assembly of a wheel module.

A wheel module (Fig. 3) consists of a DC motor with encoders, a double roller omni wheel, and a motor driver. The three wheel modules of the Robotont are designed to be easily replaceable and maintainable: after unfastening two screws, the module can slide out of the main chassis. The chassis of the wheel module is made of polycarbonate.

**Fig. 4:**
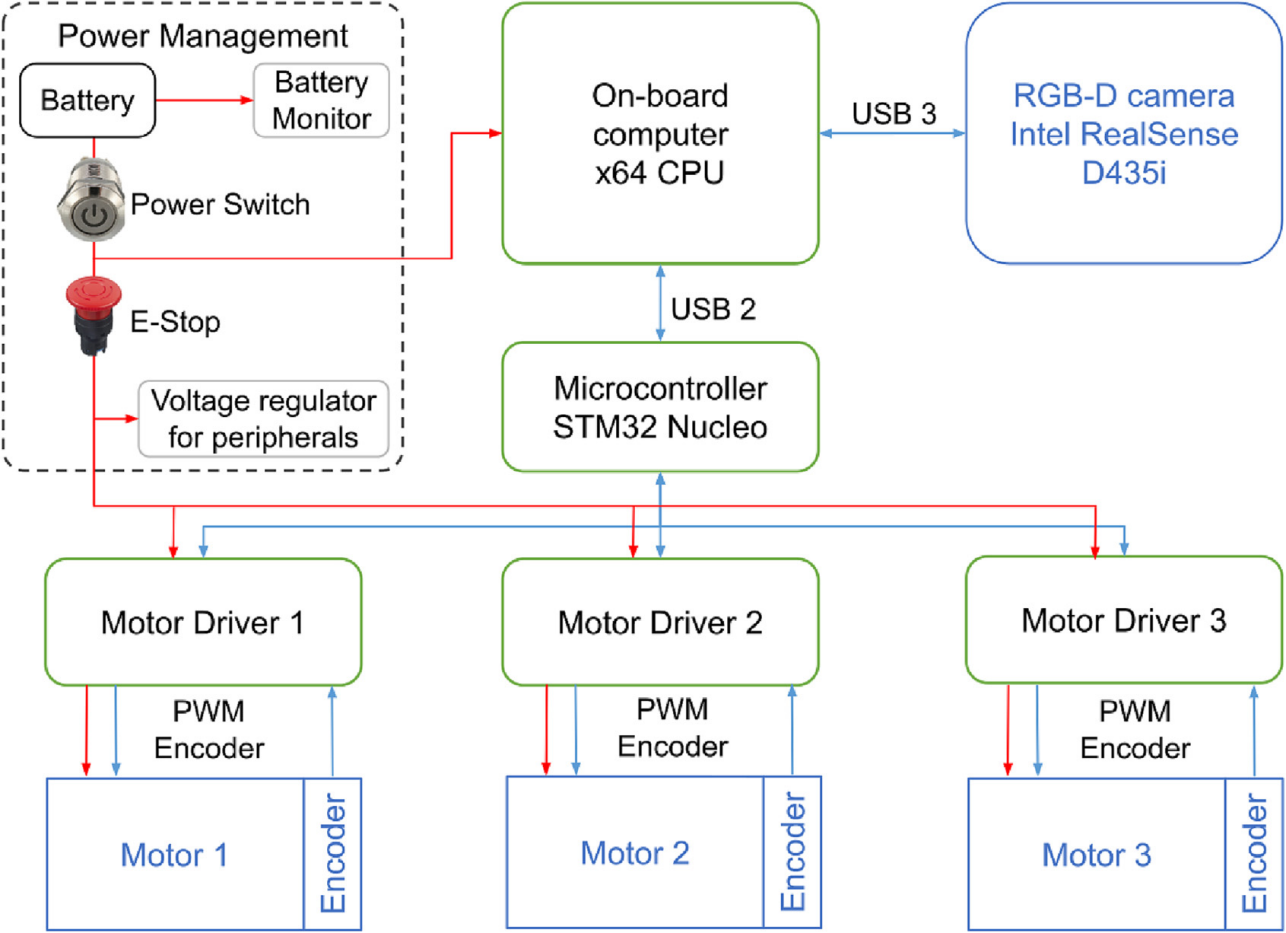
Connection architecture of Robotont. The red lines represent power delivery whereas the blue lines denote data communication. (For interpretation of the references to color in this figure legend, the reader is referred to the web version of this article.)

The buttons for power and the emergency stop (E-Stop) are integrated into the top plate of the main chassis for easy access and safety (Fig. 2). Furthermore, the top plate of the chassis has an opening to expose to the user the GPIO pins of the electronics board with the STM32 microcontroller (Fig. 2, Fig. 4). The onboard computer is also mounted on top of the chassis to expose its communication ports for additional components and debugging.

To ensure an even mass distribution on the wheels, the battery and the onboard computer - as the heaviest components - are positioned at the center of the robot (Fig. 2).

A lightweight camera mount (Fig. 2) is attached to the robot with butterfly nuts to make it detachable for storage and transportation. The performance of vision and depth sensor systems is often unstable in close proximity to the device. By elevating and moving the depth camera to the back of the robot we ensure that objects right in front of the robot are within the field of view of the sensor.

For additional mechanical modularity, there are rails with the width of wheel modules at the front of the robot chassis for sliding in and attaching a custom-purpose module (Fig. 2). Also, a polycarbonate tray is mounted over the onboard computer to create space for more components or payload (up to 10 kg).

### Electronics

Robotont is centrally controlled via its onboard computer which connects to three subsystems: power management circuitry, Intel RealSense RGB-D camera, and an STM32 NUCLEO-L476RG development board. The architecture of electronics is outlined in Fig. 4.

The STM32 NUCLEO-L476RG development board is responsible for all the low-level computation in the Robotont and serves as a gateway to the three wheel modules. Each wheel module consists of a DC motor, an encoder attached to the motor shaft, and a custom motor driver board^1^. The driver board hosts an MC33886 H-bridge chip that receives a pulse width modulated (PWM) control signal from the STM32 microcontroller and amplifies the signal for the motor. The driver board also provides protective circuitry for the H-bridge chip as well as includes connectors for convenient assembly and maintenance.

Based on the encoders, the STM32 microcontroller calculates the wheel and robot velocities, provides odometry by integrating the velocities and generates control signals for the motor drivers. A closed loop PID-controller implementation in the STM32 microcontroller helps to keep the robot at a desired heading. The data communication and power delivery from the robot's onboard computer to the microcontroller is accomplished via a USB 2.0 bus.

Robotont’s power supply is a 4-cell lithium polymer battery that connects to a power management board^2^. This board is responsible for delivering power to the wheel motor controllers as well as to the onboard computer. The power management board allows for turning the whole platform ON or OFF from a single power switch. For additional safety, an E-Stop switch is introduced to cut off power to the wheel controllers.

The onboard computer can be powered either from the battery or an AC adapter. Using power from the AC adapter avoids excessive draining of the battery when working with the robot’s onboard computer for a longer period of time.

Robotont can be extended by interfacing additional devices to the unused USB 3.0 ports of the onboard computer. One rear port and two front ports are available. The platform can also be extended via the exposed GPIO header of the STM32 Nucleo microcontroller that features a variety of communication standards including UART, SPI, I2C, and CAN. The power delivery to external devices is solved through an adjustable voltage regulator - a separate module that is built on an XL4015 chip and can be accessed from the front side of the robot. The module supports loads up to 5 A and its voltage can be set anywhere from 1.25 V up to the battery voltage. The module is connected in parallel with the motor controllers and is thus also cut off by the E-Stop.

### Software

**Fig. 5:**
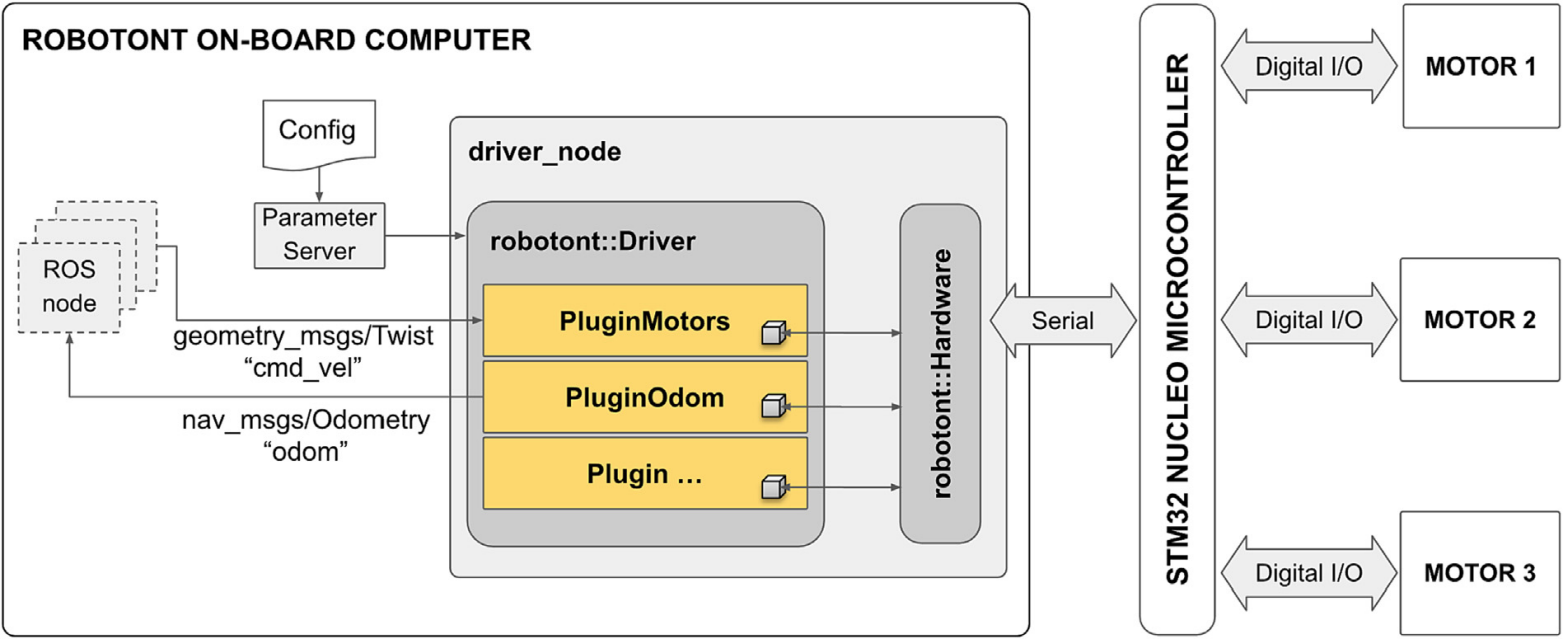
The software flowchart.

The software system for Robotont is designed to leverage the diverse set of capabilities offered by ROS. For that reason, at the heart of the software architecture lies the robotont_driver, a ROS package that contains the driver_node (Fig. 5). The driver_node serves as a gateway between high-level ROS-based functionality and the low-level microcontroller-operated motors and sensors.

As a mobile ground robot, it is paramount that the software of Robotont is compatible with ROS Navigation [16] for attaining autonomous behavior (i.e. 2D SLAM and motion planning). For integration, the ROS Navigation requires a cmd_vel/odom interface [17], i.e. the ability to receive velocity messages and send back odometry data. Thus, the driver_node of Robotont subscribes to the topic of “cmd_vel” and publishes on the topic of “odom” to provide a suitable interface (Fig. 5). At the same time, the driver_node maintains bidirectional serial communication with the microcontroller. The computation of individual motor speeds from the input velocity command as well as the odometry based on wheel encoders is handled by the firmware on the microcontroller. The software architecture of the driver_node makes use of C++ plugins to offer a modular framework for adding new functionality to the driver.

**Fig. 6:**
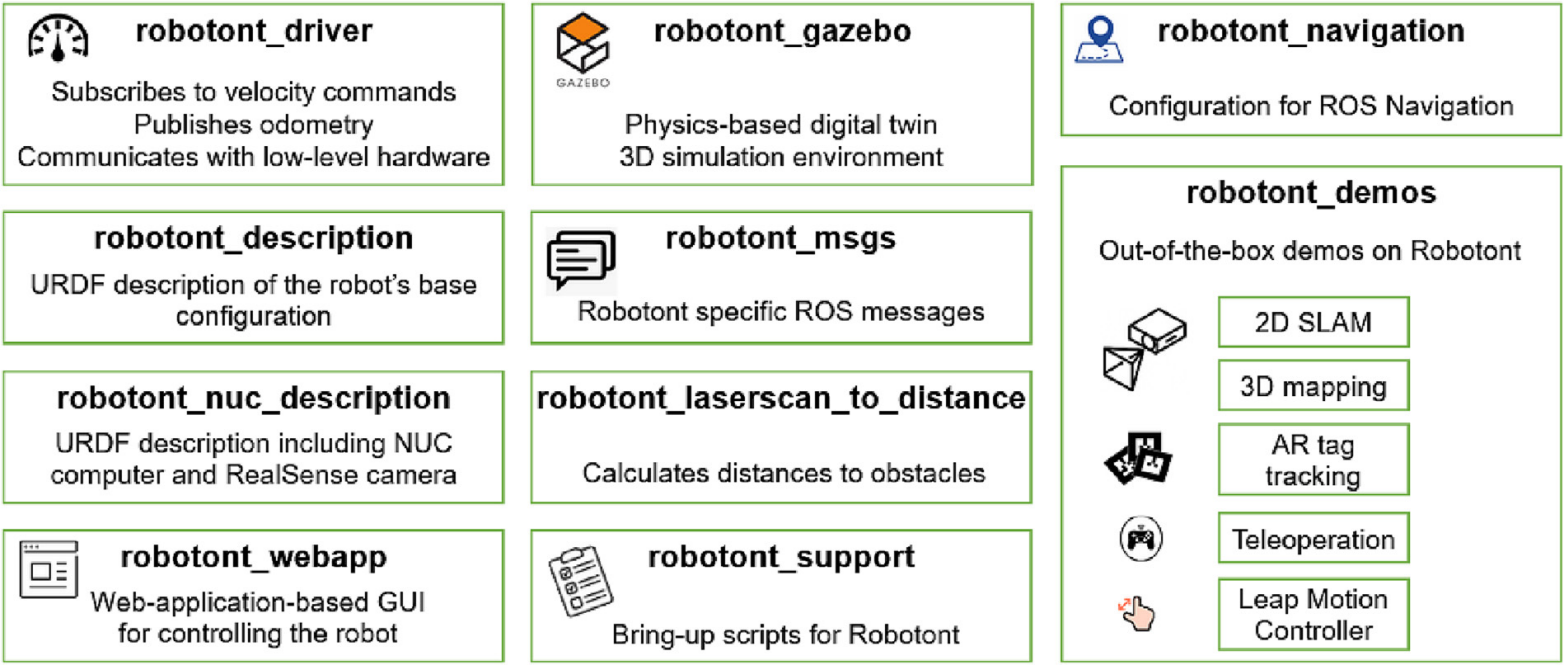
The organization of Robotont software.

The entire Robotont software stack and its documentation is available on GitHub (github.com/robotont). In adherence with ROS conventions [18], all the software is organized into functional ROS packages. The main packages and their functionality is listed in Fig. 6.

### Digital twin

**Fig. 7:**
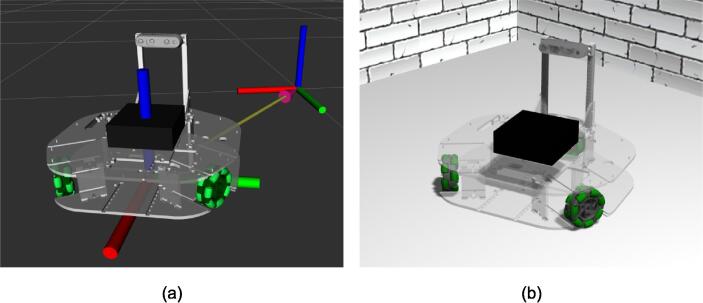
Digital twins of Robotont. (a) RViz visualization of fake controller-based digital twin with base_footprint and odom frames visualized; (b) physics-based Gazebo simulation.
2) a physics-based Gazebo simulation (Fig. 7-b).

In addition to the physical robot platform, there are two ROS-compatible digital twins of the Robotont:

1) a lightweight fake driver-based visualization (Fig. 7-a) and

These digital twins offer a software-based cost-effective alternative to using Robotont as an educational tool for learning ROS and robotics when access to the physical platform is limited or not possible.

#### Fake controller-based digital twin

In ROS, a fake controller is a node that listens to any control commands issued by a higher level software to the hardware and provides correctly formatted feedback the higher level software may require from the hardware. For mobile robots, the most straightforward form of a fake controller subscribes to the velocity commands and, in return, publishes odometry data. For Robotont, the fake controller is part of the robotont_driver package (Fig. 6) and it integrates the received velocity commands over time to provide fake but ideal odometry. Positional change of the robot can be visualized in RViz relative to the odom frame (Fig. 7-a).

#### Physics-based Gazebo simulation

**Fig. 8:**
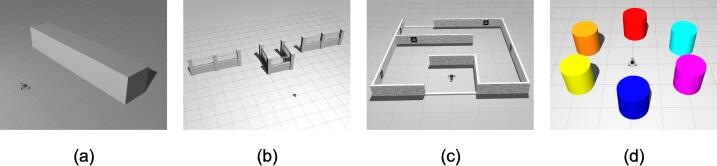
Gazebo worlds for (a) developing a two-state controller for obstacle avoidance; (b) learning to drive around an obstacle; (c) navigating a maze with or without fiducial markers; (d) implementing color-based blob detection to command the robot.

A more realistic physics-based digital twin is created for open-source robotics simulation software Gazebo and it is contained within the robotont_gazebo package (Fig. 6). Gazebo-based simulation allows testing the robot programming in scenarios where context awareness such as collision avoidance or object tracking is crucial. The Gazebo support enables Robotont to be placed into the multitude of existing simulated worlds. A set of minimalistic worlds have been created within robotont_gazebo to quickstart Robotont simulation and serve as characteristic example environments (Fig. 8) for learning fundamental concepts of robotics (e.g., obstacle and color detection, mapping, and localization). ROS and Gazebo have a native compatibility and Gazebo-based simulation is typically combined with RViz visualization when developing or learning ROS [17].

## Design file summary

**Fig. 9:**
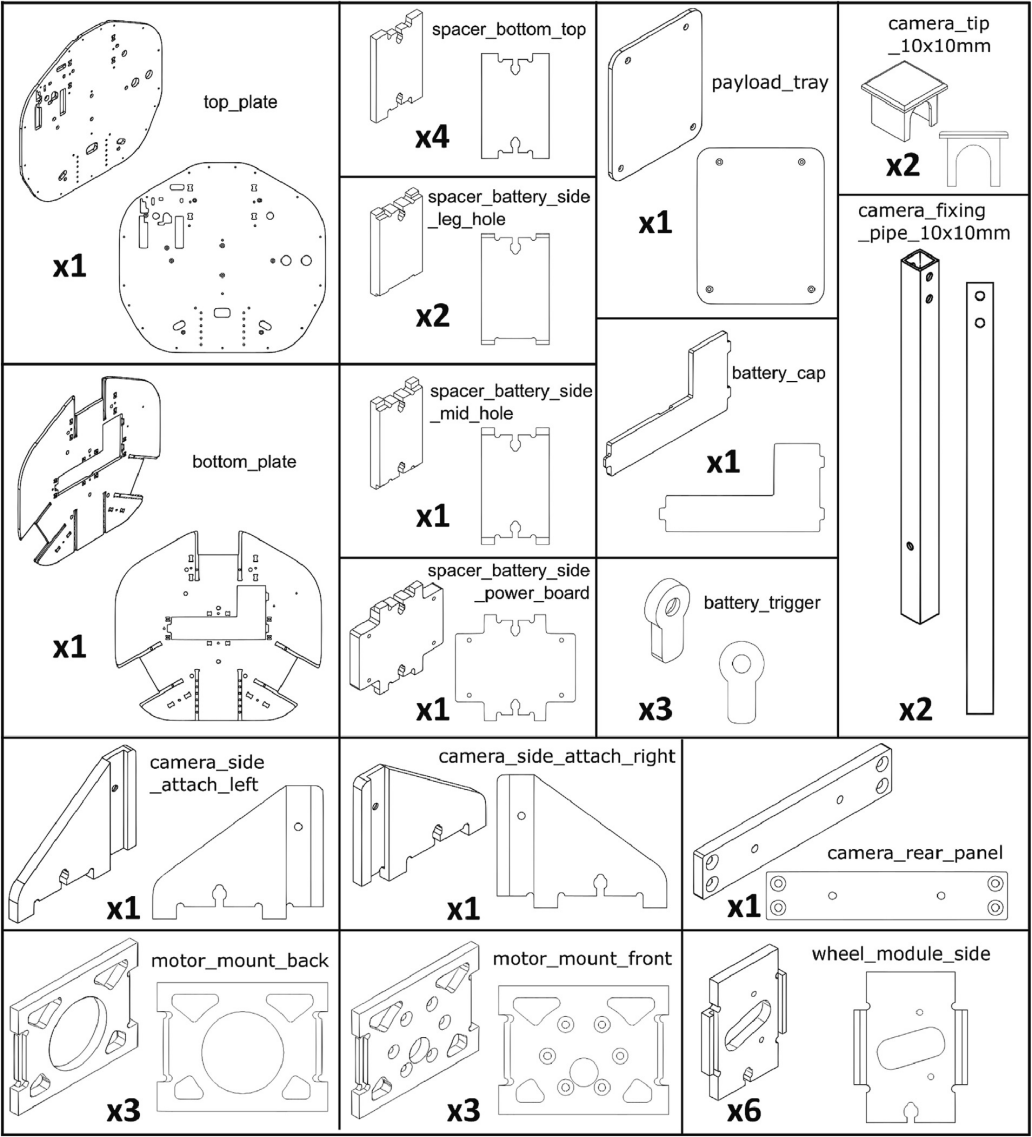
Graphical design file summary.

Table 1 and Fig. 9 provide an overview of all the mechanical components that need to be manufactured for assembling Robotont.

**Table 1:** Design file summary.

Design file name	File type	Open-source license	Location of the file
top_plate	STEP file	CERN-OHL-P-2.0	https://doi.org/10.5281/zenodo.7897546
bottom_plate	STEP file	CERN-OHL-P-2.0	https://doi.org/10.5281/zenodo.7897546
spacer_bottom_top	STEP file	CERN-OHL-P-2.0	https://doi.org/10.5281/zenodo.7897546
spacer_battery_side_leg_hole	STEP file	CERN-OHL-P-2.0	https://doi.org/10.5281/zenodo.7897546
spacer_battery_side_mid_hole	STEP file	CERN-OHL-P-2.0	https://doi.org/10.5281/zenodo.7897546
spacer_battery_side_power_board	STEP file	CERN-OHL-P-2.0	https://doi.org/10.5281/zenodo.7897546
motor_mount_back	STEP file	CERN-OHL-P-2.0	https://doi.org/10.5281/zenodo.7897546
motor_mount_front	STEP file	CERN-OHL-P-2.0	https://doi.org/10.5281/zenodo.7897546
wheel_module_side	STEP file	CERN-OHL-P-2.0	https://doi.org/10.5281/zenodo.7897546
battery_cap	STEP file	CERN-OHL-P-2.0	https://doi.org/10.5281/zenodo.7897546
battery_trigger	STEP file	CERN-OHL-P-2.0	https://doi.org/10.5281/zenodo.7897546
camera_side_attach_left	STEP file	CERN-OHL-P-2.0	https://doi.org/10.5281/zenodo.7897546
camera_side_attach_right	STEP file	CERN-OHL-P-2.0	https://doi.org/10.5281/zenodo.7897546
camera_rear_panel	STEP file	CERN-OHL-P-2.0	https://doi.org/10.5281/zenodo.7897546
camera_fixing_pipe_10x10mm	STEP file	CERN-OHL-P-2.0	https://doi.org/10.5281/zenodo.7897546
camera_tip_10x10mm	STEP / STL files	CERN-OHL-P-2.0	https://doi.org/10.5281/zenodo.7897546
payload_tray	STEP file	CERN-OHL-P-2.0	https://doi.org/10.5281/zenodo.7897546

## Bill of materials summary

A comprehensive bill of materials can be found in the Supplemental material S1 while the most up-to-date version of it can be found in the GitHub for Robotont. In this section, an overview of key mechanical (Table 2) and electrical (Table 3) components are listed.

### Mechanics

**Table 2:** Bill of materials for the mechanical components.

Designator	Component	Number	Cost per unit -EUR	Total cost - EUR	Source of materials	Material type
Table 1	Main chassis and wheel modules, camera and battery attachment, addons	1	205	205	https://github.com/robotont/robotont-mechanics/tree/Ver-2.1/Production	Polycarbonate; aluminium, PLA
	Pololu 37Dx68L motor	3	47	141	https://www.pololu.com/product/4751	non-specific
	Vex Robotics wheel	3	12	36	https://www.vexrobotics.com/wheels.html	non-specific
	Fasteners, spacers and couplings	116	0.08	10	Supplemental material S1	zinc coated; copper

### Electronics

**Table 3:** Bill of materials for the electronics.

Designator	Component	Number	Cost per unit - EUR	Total cost - EUR	Source of materials
Onboard computer ×64 CPU	Intel NUC with at least i5 processor	1	600	600	https://www.intel.com/content/www/us/en/products/details/nuc/mini-pcs/products.html
Microcontroller STM32 Nucleo	STM32 NUCLEO-L476RG development board	1	30	30	https://www.tme.eu/details/nucleo-l476rg
	Connector shield for NUCLEO-L476RG	1	2	2	https://doi.org/10.5281/zenodo.7897546https://github.com/robotont/robotont-electronics-nucleo-shield
	Motor driver board	3	25	75	https://doi.org/10.5281/zenodo.7897546https://github.com/robotont/robotont-electronics-driver-board
	Power management board	1	10	10	https://doi.org/10.5281/zenodo.7897546https://github.com/robotont/robotont-electronics-power-management-board
	Voltage regulator for peripherals	1	3	3	Supplemental material S1
Power supply	Battery – 4 cell LiPo at least 4000 mAh	1	50	50	https://hobbyking.com/en_us/turnigy-high-capacity-5200mah-4s-12c-multi-rotor-lipo-pack-w-xt60.html
	RGB-D camera Intel RealSense D435i	1	330	330	https://www.intelrealsense.com/depth-camera-d435i
	Camera connection cable - USB-C 90 deg to USB-C	1			
	Mini USB 90 deg to USB-A for STM32 NUCLEO-L476RG development board	1			
	E-Stop button	1	7	7	
	Power button	1	2	2	
	Custom cabling				Fig. 12, Fig. 13, Fig. 14; Supplemental S1

## Build instructions

**Fig. 10:**
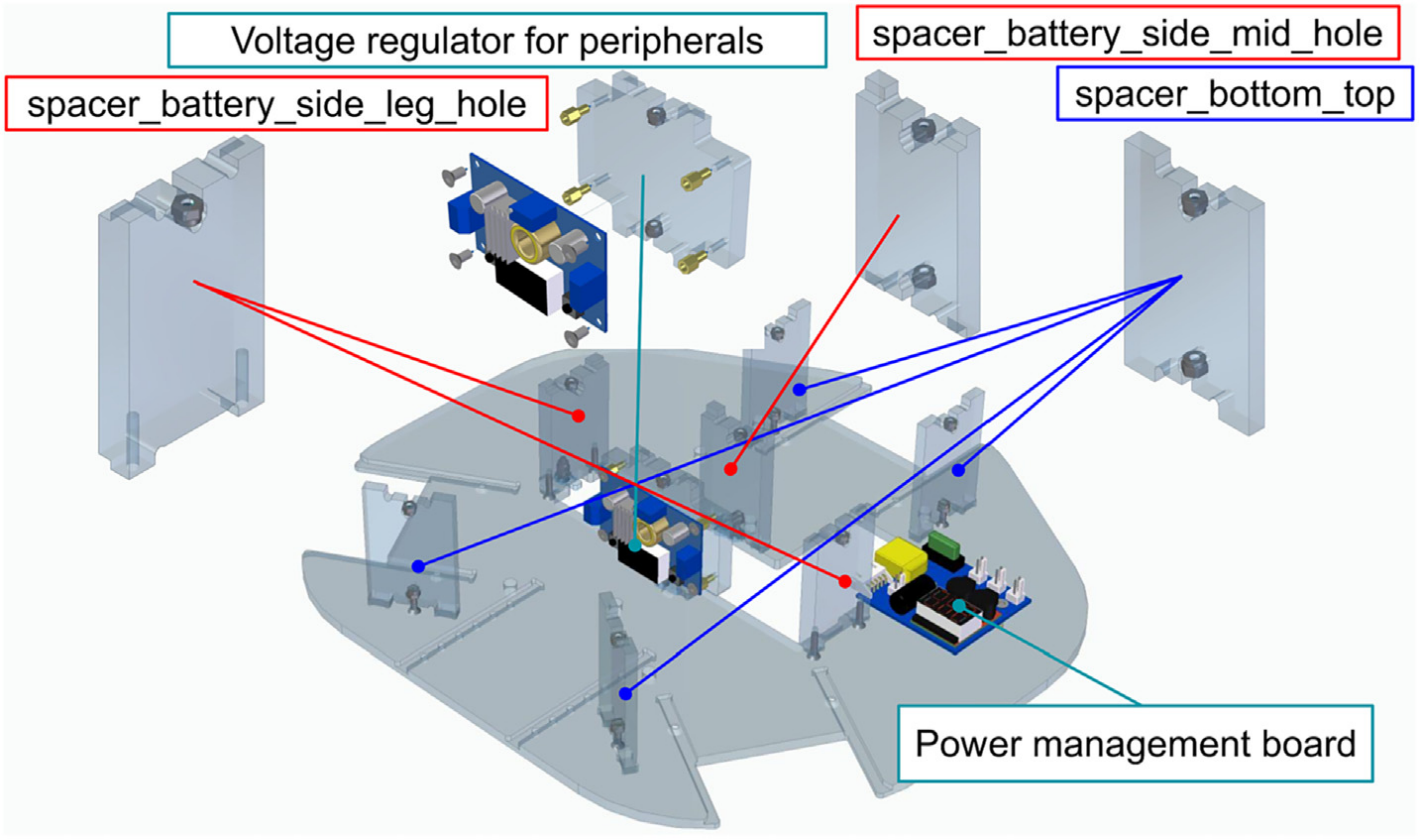
Assembly on the bottom plate.

In this section we outline the recommended flow of assembling the Robotont mobile robot. The comprehensive overview of build instructions is provided in supplemental material S2. As the first step, we take the bottom plate and mount different spacers and two circuit boards to it (Fig. 10).

**Fig. 11:**
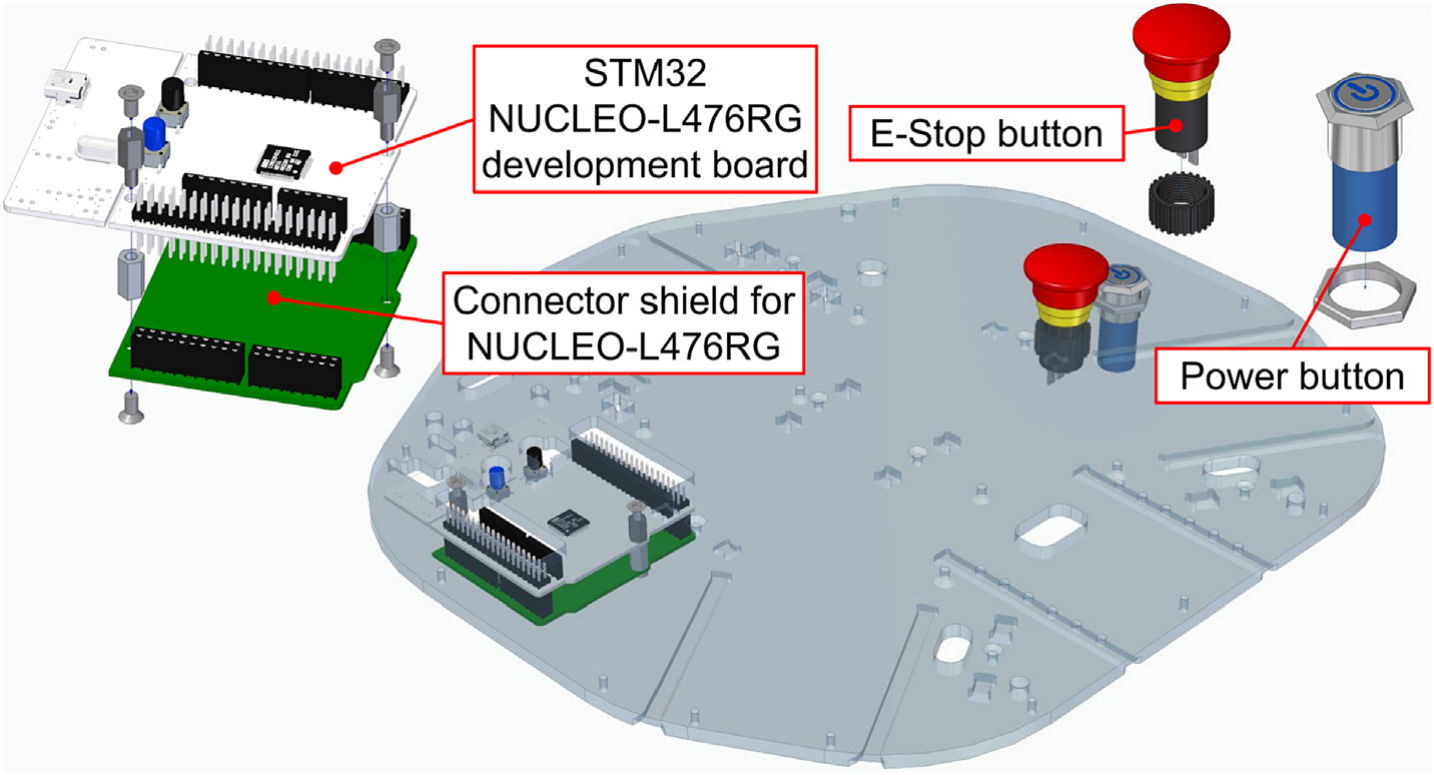
Assembly on the top plate.

Next we attach the main electronics controller and the buttons for power and E-stop to the top plate (Fig. 11).

**Fig. 12:**
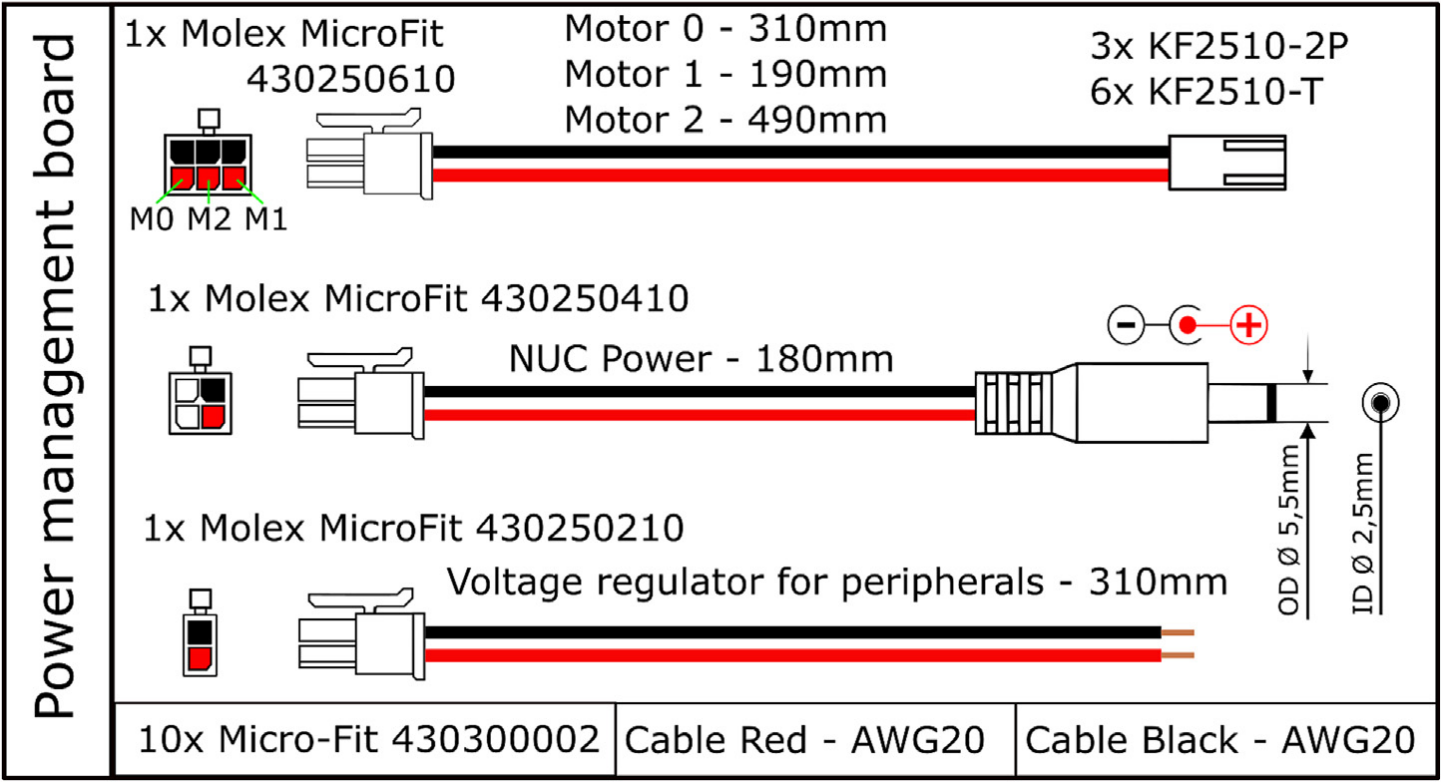
Power cables.

**Fig. 13:**
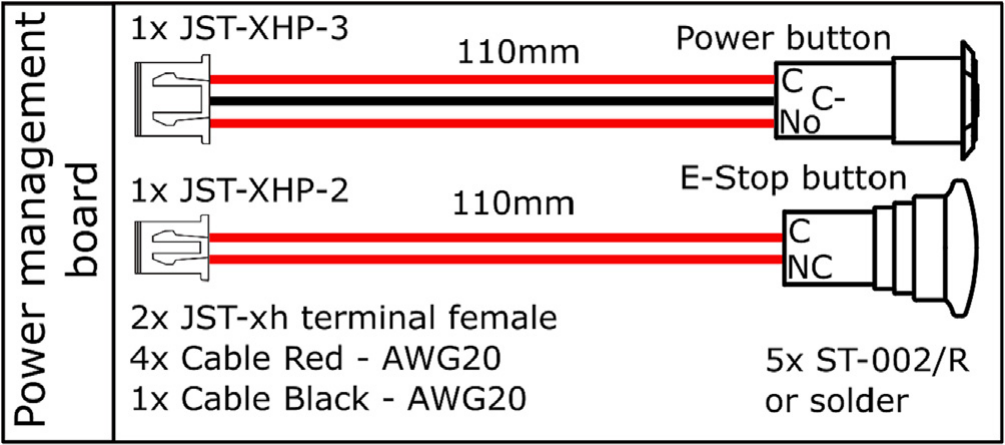
Power and E-Stop button assemblies.

**Fig. 14:**
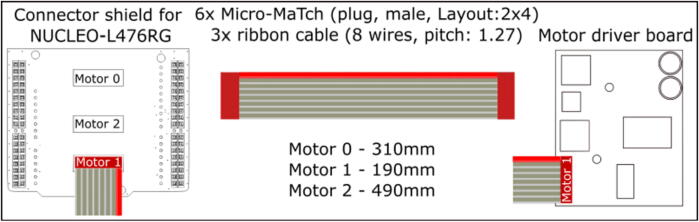
Ribbon cable assemblies for connecting motor driver boards.

Prepare the cabling as detailed in Fig. 12, Fig. 13, Fig. 14 and connect them accordingly within the main chassis of the Robotont.

**Fig. 15:**
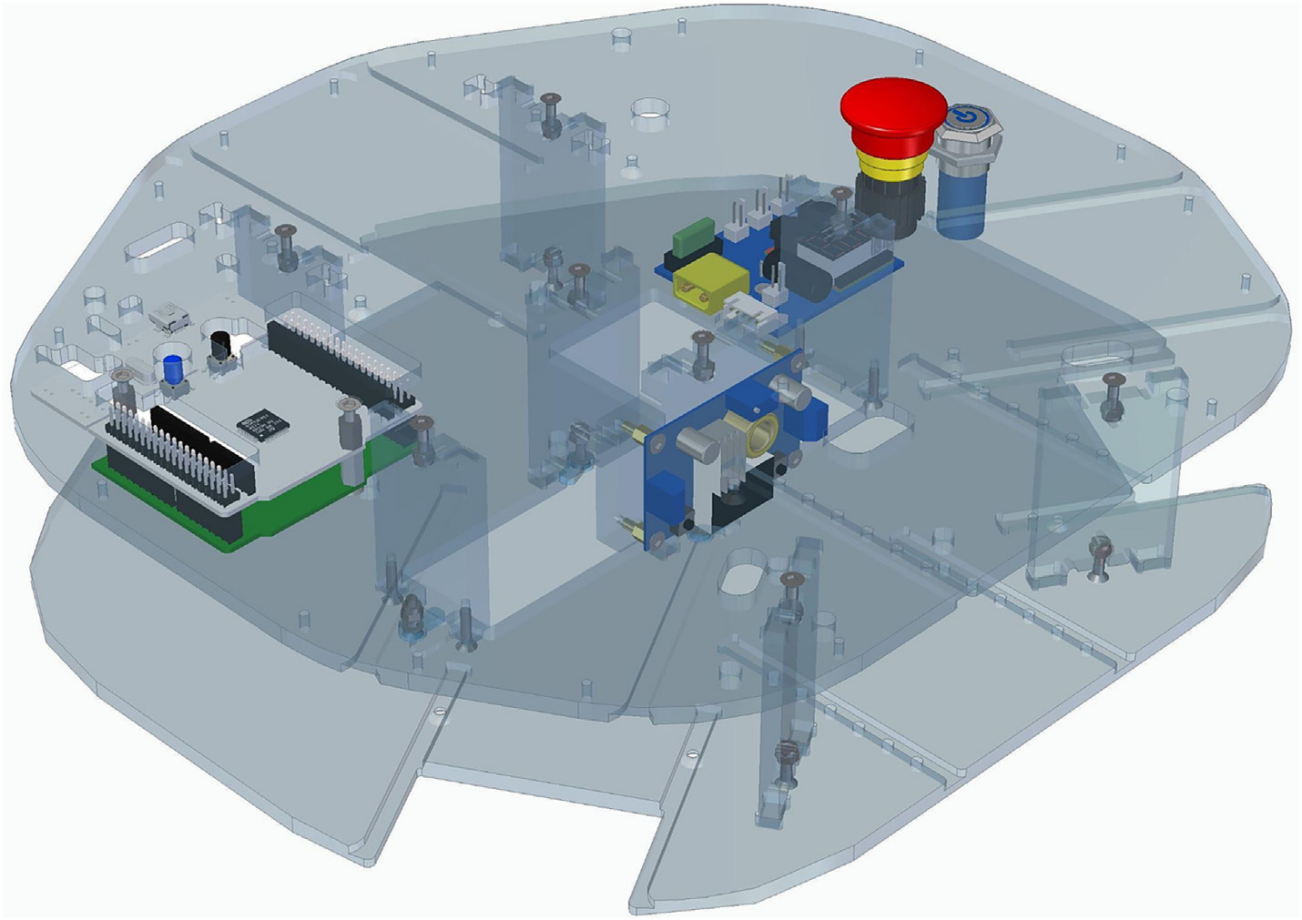
Assembly of top and bottom plates to form the main chassis.

We can now mount the top plate on the bottom plate as depicted in Fig. 15.

**Fig. 16:**
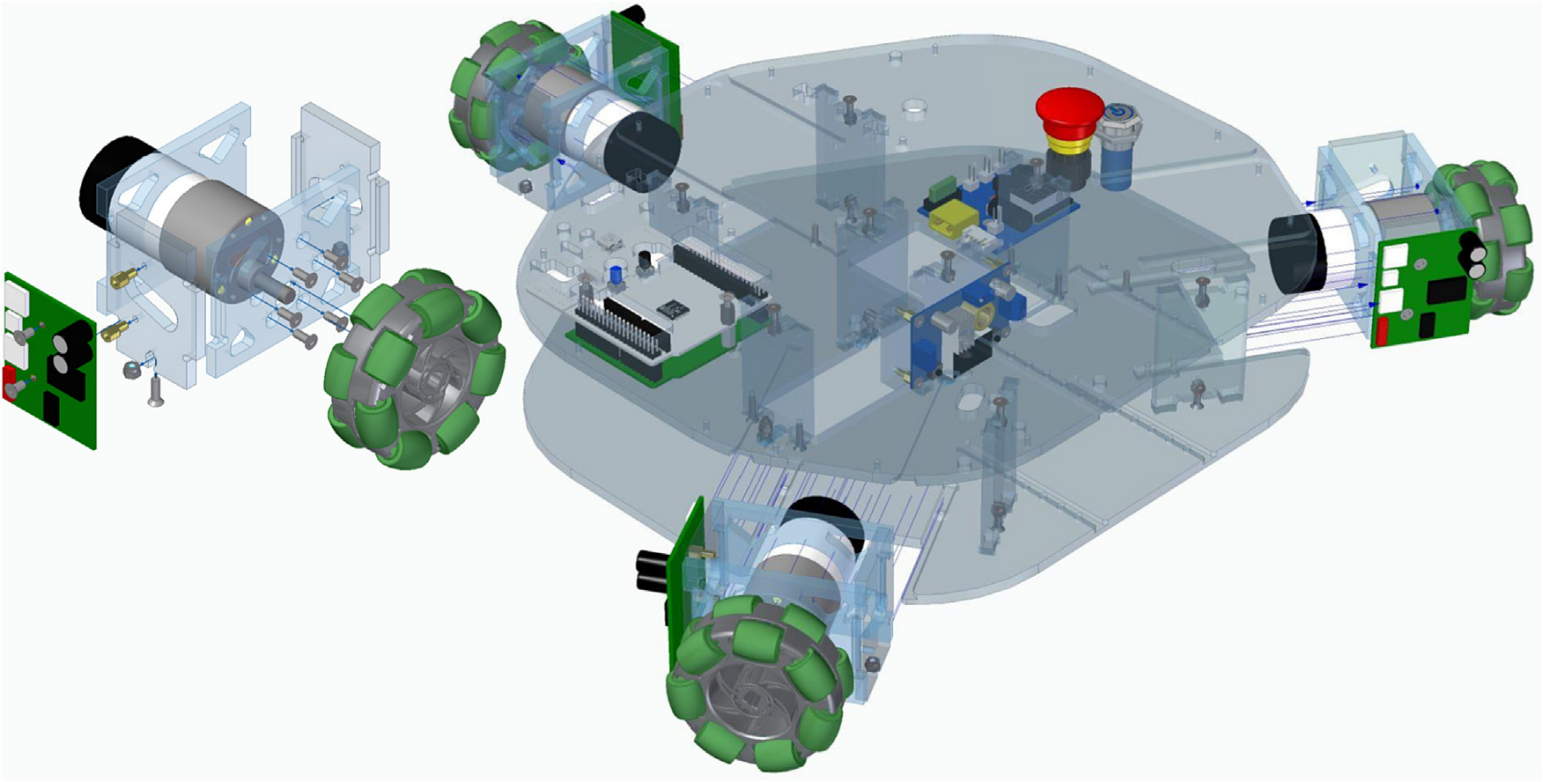
Assembly of wheel modules.

We assemble three wheel modules as depicted in Fig. 3 and slide them into the main chassis as shown in Fig. 16.

**Fig. 17:**
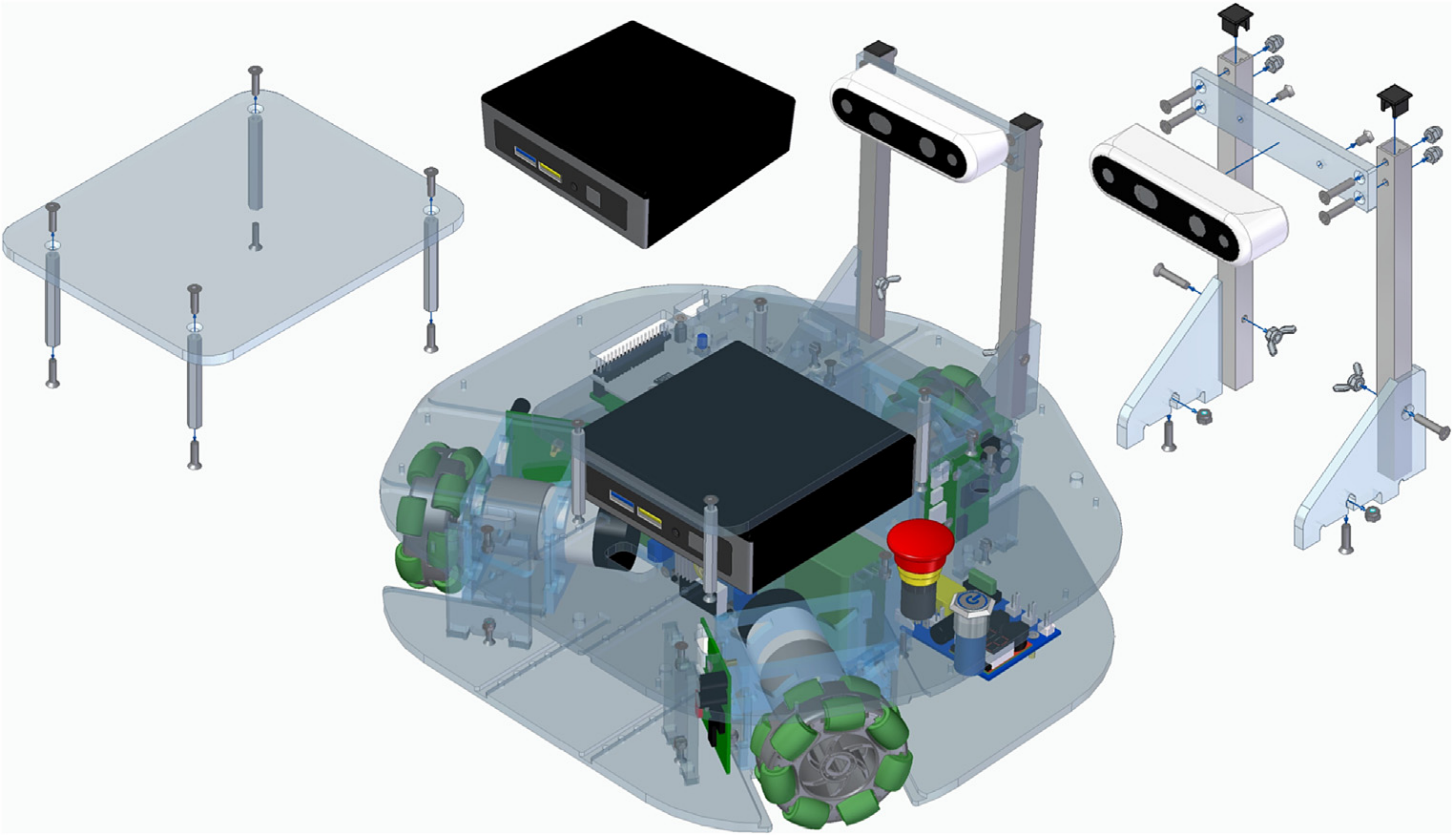
Mounting the onboard computer, camera and the payload tray to the top plate.

As a final step, we attach the onboard computer, camera, and payload tray (Fig. 17).

## Operation instructions

### Setting up the software

To set up Robotont, it is needed to install software on the microcontroller and on the onboard computer. Both procedures are fairly straightforward and supported by automated scripts.

#### Uploading firmware to the STM32 NUCLEO microcontroller

The STM32 NUCLEO-L476RG is split into two separate sections - the development board with a microcontroller and an ST-Link embedded programmer. Due to the onboard programming capability, the firmware of the controller can be uploaded by simply copying the binary file to the programmer which shows up as a USB mass storage device when connected to a computer.

*Prerequisites:*

1) Computer with an Internet connection and unused USB port.

*Instructions:*

1) Connect the STM Nucleo board to the computer via a USB cable.
2) Navigate to the releases section in the robotont-firmware^3^ repository website and download the bin-file with the latest firmware.
3) Copy the downloaded bin-file to the removable storage drive that appears after connecting the STM Nucleo board.
4) Wait until the LED on the STM Nucleo board stops flashing.
5) The firmware is uploaded to the microcontroller and is ready to use.

#### Installing Linux, ROS, and Robotont software

To set up the onboard computer of Robotont, we first have to install the Ubuntu operating system and then run automated configuration scripts available in the robotont-setup^4^ repository. The scripts will automatically install and configure multiple components including ROS with its build tools and dependencies, set up catkin workspace with Robotont packages, startup services, etc. It also configures the robot as an access point The automation is written for the Ansible Automation Platform [19], which allows the setup to be performed either locally on a single robot or remotely via SSH connection for multiple robots simultaneously. The following instructions demonstrate the local installation.

*Prerequisites:*

1) Empty USB stick (at least 8 GB)
2) Computer mouse
3) Computer keyboard
4) Display with an HDMI connector
5) Internet connection (WiFi or ethernet)

*Instructions:*

1) Create a bootable Ubuntu USB stick and connect it to the onboard computer of the robot
2) Power on the computer and enter the boot menu (for Intel NUC press F10 key during boot)
3) In the boot menu, select USB as the boot device
4) Follow the Ubuntu installation. Use the following names to align with the tutorials:
  a) Hostname: robotont-1
  b) Username: peko
5) Download the robotont-setup repository on the robot. For that open a terminal with CTRL + ALT + T and input the following commands:
  a) sudo apt install git
  b) git clone https://github.com/robotont/robotont-setup
6) Adjust the 'hosts' file located in the 'robotont-setup/ansible' folder.
  a) Make sure that the ansible_user variable equals the value set during the Ubuntu installation (step 4b).
  b) The robot will become a WiFi access point after the configuration is finished. The network access key can be set with the wifi_pass variable.
7) Run the following command to install ansible:
  a) sudo apt install ansible
8) Change the terminal working directory to the one containing the configuration files:
  a) cd robotont-setup/ansible
9) Run ansible and enter the Ubuntu user password (set during the installation in step 4) in the BECOME field when it appears:
  a) ansible-playbook robots-local.yaml -K

After the setup has finished, the onboard computer is rebooted automatically. Should that fail, reboot manually and your robot is ready.

### Working with the robot

There are multiple ways of working with Robotont after it has been properly set up. In this section three characteristic methods are explained: webapp-based teleoperation; deploying a distributed ROS system to run across Robotont onboard computer and the user’s computer; and creating an SSH tunnel to Robotont onboard computer.

#### Webapp-based teleoperation

The webapp-based teleoperation enables straightforward control of the Robotont. The operator can use a web browser to gain access to an application which allows direct control over.

*Prerequisites:*

1) Robotont set up as described in section “Setting up the software”.
2) WiFi-capable device (e.g., a laptop computer or smartphone) with a web browser.

*Instructions:*

1) Power ON the Robotont.
2) Wait for a Robotont-hosted wireless network to appear and connect to it.
3) Open a web browser and insert the robot’s IP address or hostname to the address bar.
4) A web page loads showing the controls for the robot.

#### Distributed ROS across the onboard and user computers

ROS is designed by keeping distributed computing in mind. Therefore, a developer or learner of ROS is often encouraged to configure their ROS to run across multiple computers, e.g., the robot’s onboard computer and the user’s computer. The examples in this subsection are given assuming the following network configuration:

**Table:** 

	hostname	IP-address
user’s computer	laptop-1	192.168.200.101
robot onboard computer	robotont-1	192.168.200.1

*Prerequisites:*

1) Robotont set up as described in section “Setting up the software”.
2) A WiFi-capable computer with Linux and ROS that matches the Robotont’s software setup.

*Instructions:*

1) Power ON the Robotont
2) Wait for a Robotont-hosted wireless network to appear and connect to it.
3) Open a new terminal window on the user’s computer.
4) Verify that the user computer and the robot are able to ping each other via hostnames
  a) On the user's computer run: ping robotont-1
5) Use the SHH client to make an SHH connection to the IP address of the robot and run:
  a) On the robot onboard computer: ping laptop-1
6) If the machines are not able to reach each other, add the name and IP pairs to /etc/hosts
  a) On the robot onboard computer: 192.168.200.101 laptop-1
  b) On the user's computer: 192.168.200.1 robotont-1
7) Set up the ROS_MASTER_URI environmental variable on the user's computer by running:
  a) export ROS_MASTER_URI https://robotont-1:11311
8) The environment in the particular terminal window of the user's computer is now configured to connect to the ROS master on the robot.
9) Optional: Validate that distributed ROS was successfully configured by running keyboard-based teleoperation on the user’s computer: rosrun teleop_twist_keyboard teleop_twist_keyboard.py

#### Creating an SSH tunnel to the onboard computer

An SSH tunnel is a very typical way of accessing another computer over a network connection.

*Prerequisites:*

1) Robotont set up as described in section “Setting up the software”.
2) A WiFi-capable computer with an SSH client software.

*Instructions:*

1) Power ON the Robotont
2) Wait for a Robotont-hosted wireless network to appear and connect to it.
3) Use the SHH client to make an SHH connection to the IP address of the robot.
4) Optional: Validate the SSH connection by running keyboard-based teleoperation on the command line of the SSH client:
  rosrun teleop_twist_keyboard teleop_twist_keyboard.py.

## Validation and characterization

Robotont is being continuously validated as an educational tool and a research platform - notable examples of such results have already been reported in a number of research publications [20], [21], [22], [23], [24], [25], [26], [27]. More specifically, this robot has been extensively used to deliver university teaching, professional training, and online courses on the topics of ROS and robotics as described in [20], [21], [22], [23]. Furthermore, Robotont has been a subject or demonstrator tool in a growing number of student theses (e.g. [28], [29], [30], [31], [32]) and a platform to validate cutting-edge robotics research about e.g. software frameworks [24] and motion-planning [25], [26], [27].

Such widespread adoption of Robotont is a testament to the technological maturity of the platform and its native ROS support. Due to the ROS support, Robotont platform can integrate software capabilities available within the ROS ecosystem. However, to streamline ease of use we have preconfigured the key capabilities associated with mobile robots (i.e. teleoperation, 2D simultaneous localization and mapping, autonomous navigation, 3D mapping, and tracking of fiducial markers) and included them with step-by-step instructions in the robotont_demos package (Fig. 6). While the reported features provide an overview of the current state of development, the list of capabilities is continuously growing. For the latest information, the reader is advised to refer to the documentation on GitHub (github.com/robotont).

## Declaration of Competing Interest

The authors declare that they have no known competing financial interests or personal relationships that could have appeared to influence the work reported in this paper.

## Footnotes

1 github.com/robotont/robotont-electronics-driver-board.

2 github.com/robotont/robotont-electronics-power-management-board.

4 github.com/robotont/robotont-setup.

## Footnotes

3 github.com/robotont/robotont-firmware/releases.
