# Supplementary material for: ROBOTONT – Open-source and ROS-supported omnidirectional mobile robot for education and research
Source: HardwareX. 2023 Jun 1;14:e00436. doi: 10.1016/j.ohx.2023.e00436 (PMC10329175; doi:10.1016/j.ohx.2023.e00436)

ROBOTONT

assembly instructions

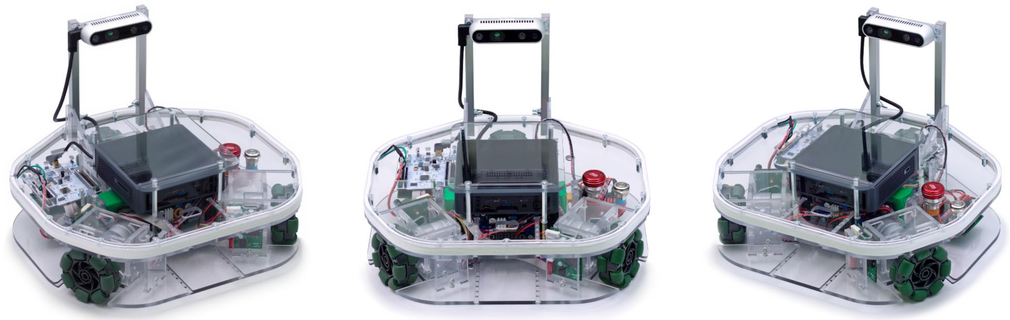

Robotont version: gen2.1
Date of the file: 2023-03-03

Table of contents

Manufacturing 3

Cabling 6

Robot assembly 10

Manufacturing

List of parts manufactured by CNC mill

Files for production: github.com/robotont/robotont-mechanics/tree/Ver-2.1

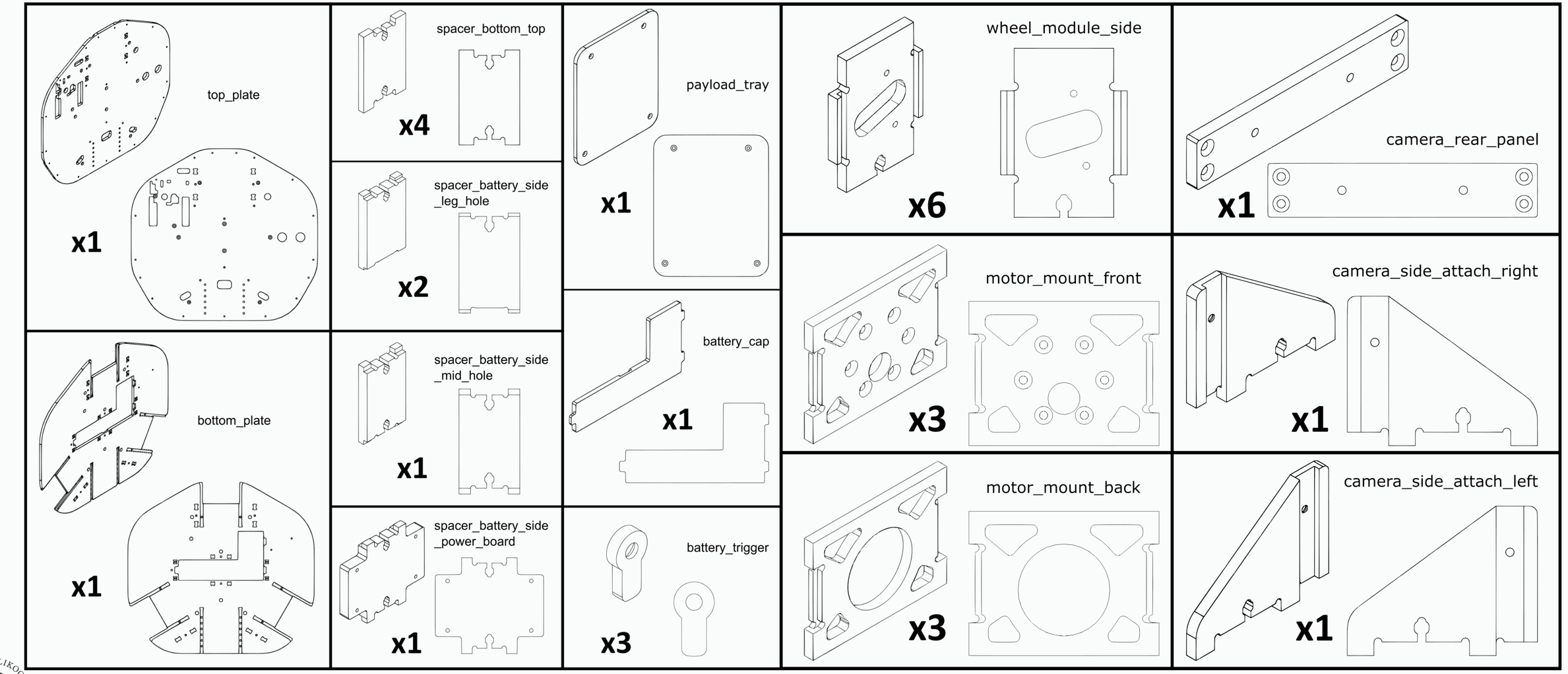

List of parts manufactured by hand tools and 3D printing

Files and drawings for production:
github.com/robotont/robotont-mechanics/tree/Ver-2.1

3D printer is used to produce the **camera_tip_10x10mm**

Hand tools and drill press are used to produce **camera_fixing_pipe_10x10mm**

2.5 mm drill is used to drill two holes into **spacer_battery_side_leg_hole** bottom legs

Holes marked with green are threaded to **M3**

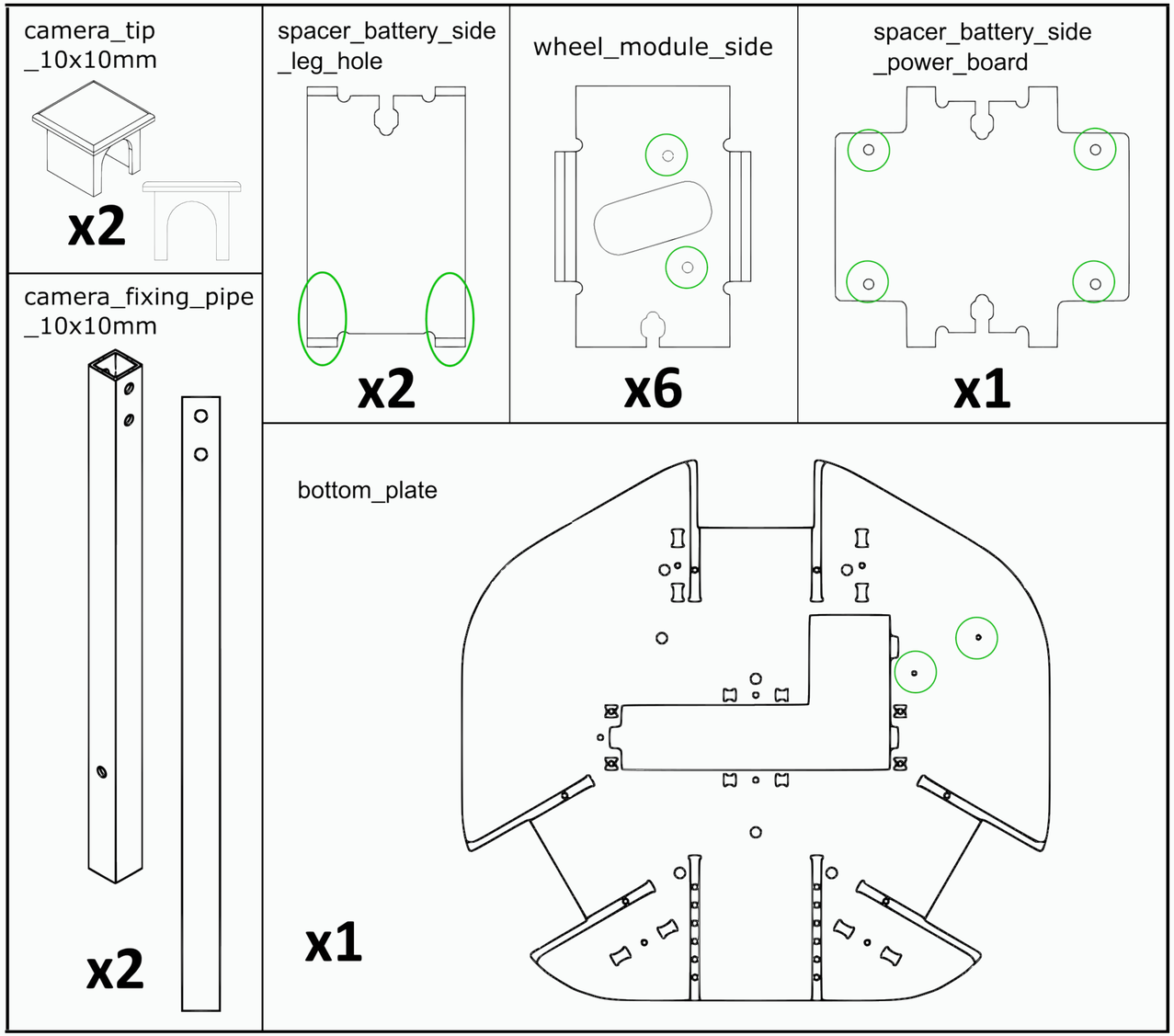

Cabling

E-Stop and Power buttons

1) Attach the terminals and housings to the “Power management board” end of cabling
2) Buttons can be either soldered or crimped with ST-002/R terminals

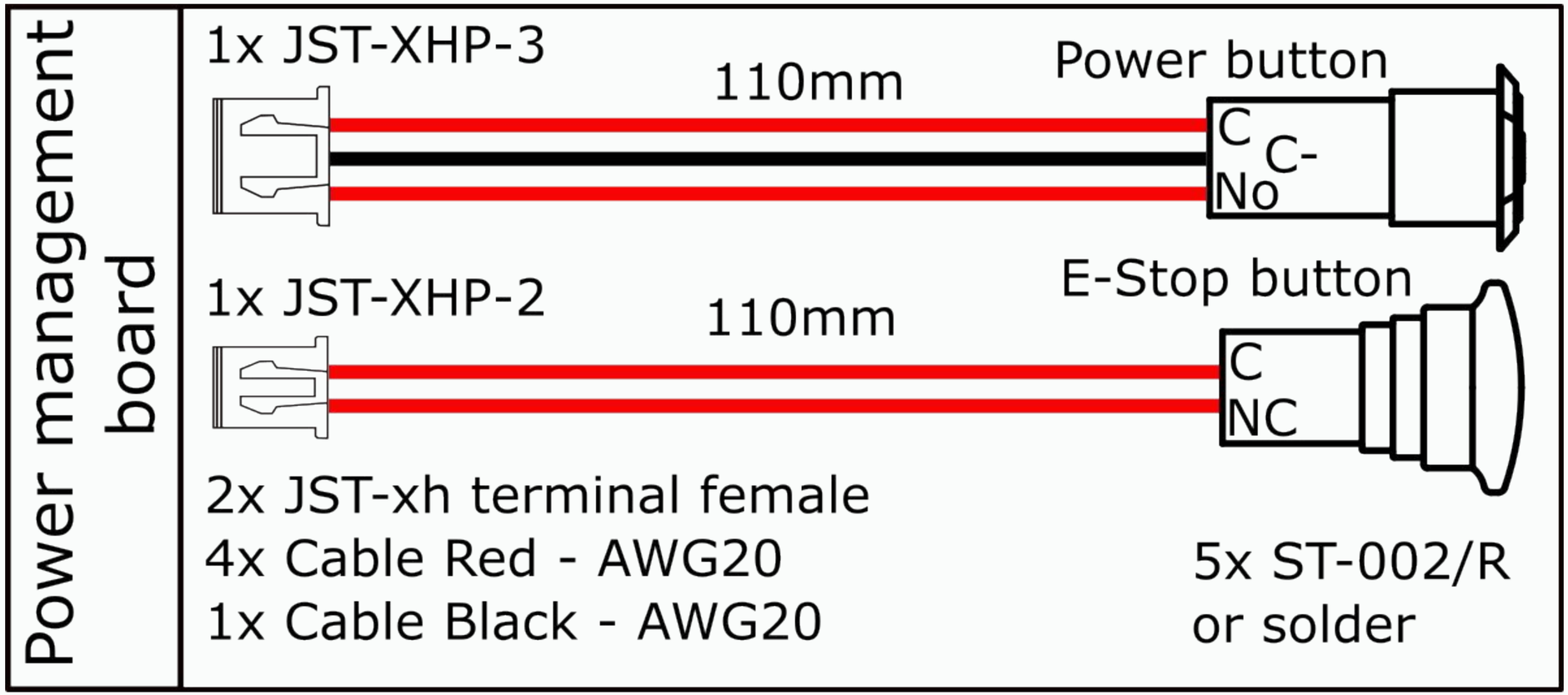

Ribbon cables from **connector shield** to **motor driver** boards

1) Use vises or a press tool to assemble the ribbon cables
2) Connect the three ribbon cables to the **connector shield** as depicted on the left.
Motor 0 and Motor 2 are connected in the same way as Motor 1.
3) The image on right shows how the ribbon cable is connected to the **motor driver board**.

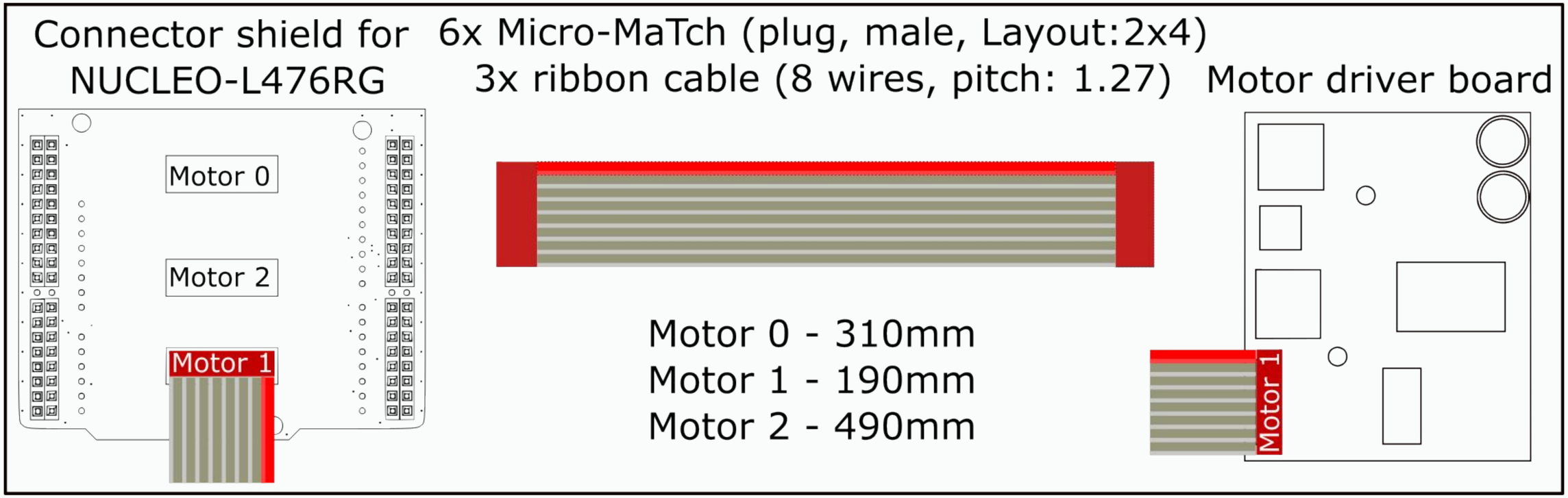

Power cables

1) Attach the terminals and housings to the “Power management board” end of cabling
2) Crimp the other end of motor cables
3) Solder the other end of NUC power cable
4) Leave the other end of the voltage regulator cable stripped or cover with ferrule

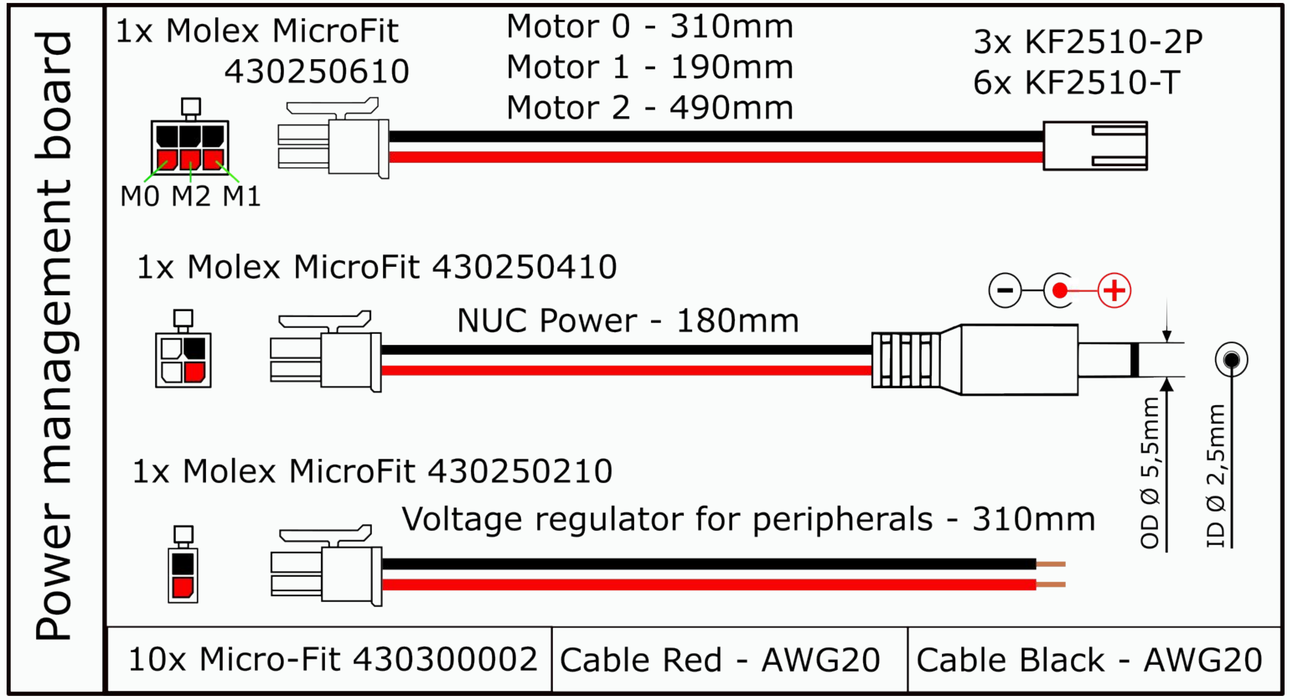

Robot assembly

Use press tools or vises to fit
M3 self-locking nuts (x14)
into spacers

spacer_battery_side_mid_hole (x1)

spacer_bottom_top (x4)

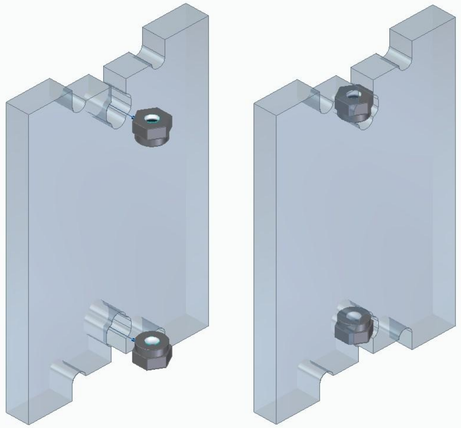

spacer_battery_side_power_board (x1)

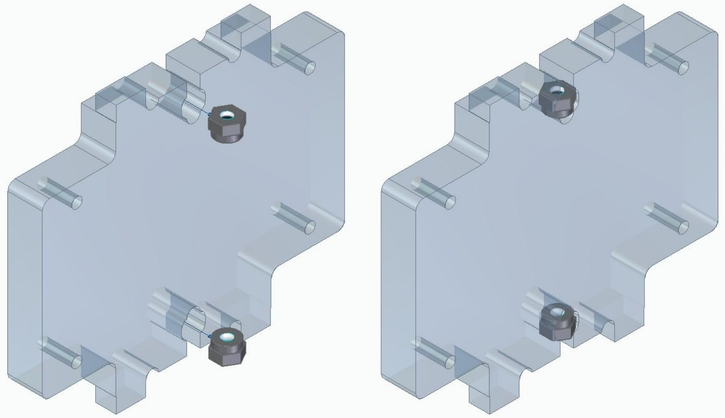

spacer_battery_side_leg_hole (x2)

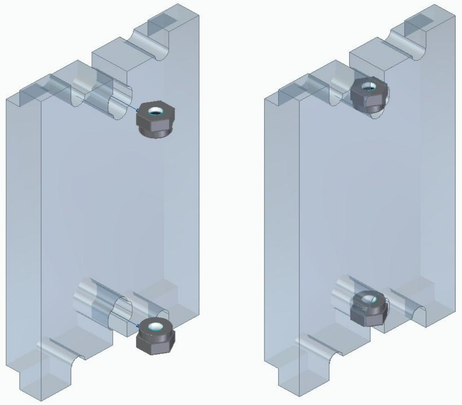

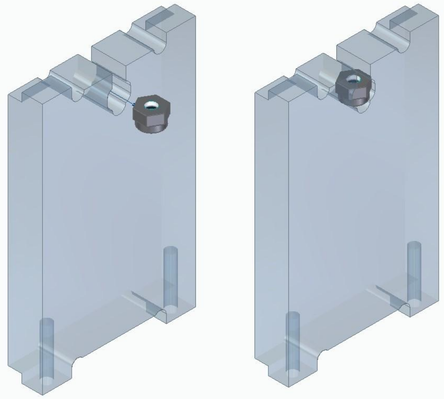

Assemble the **bottom_plate** as follows

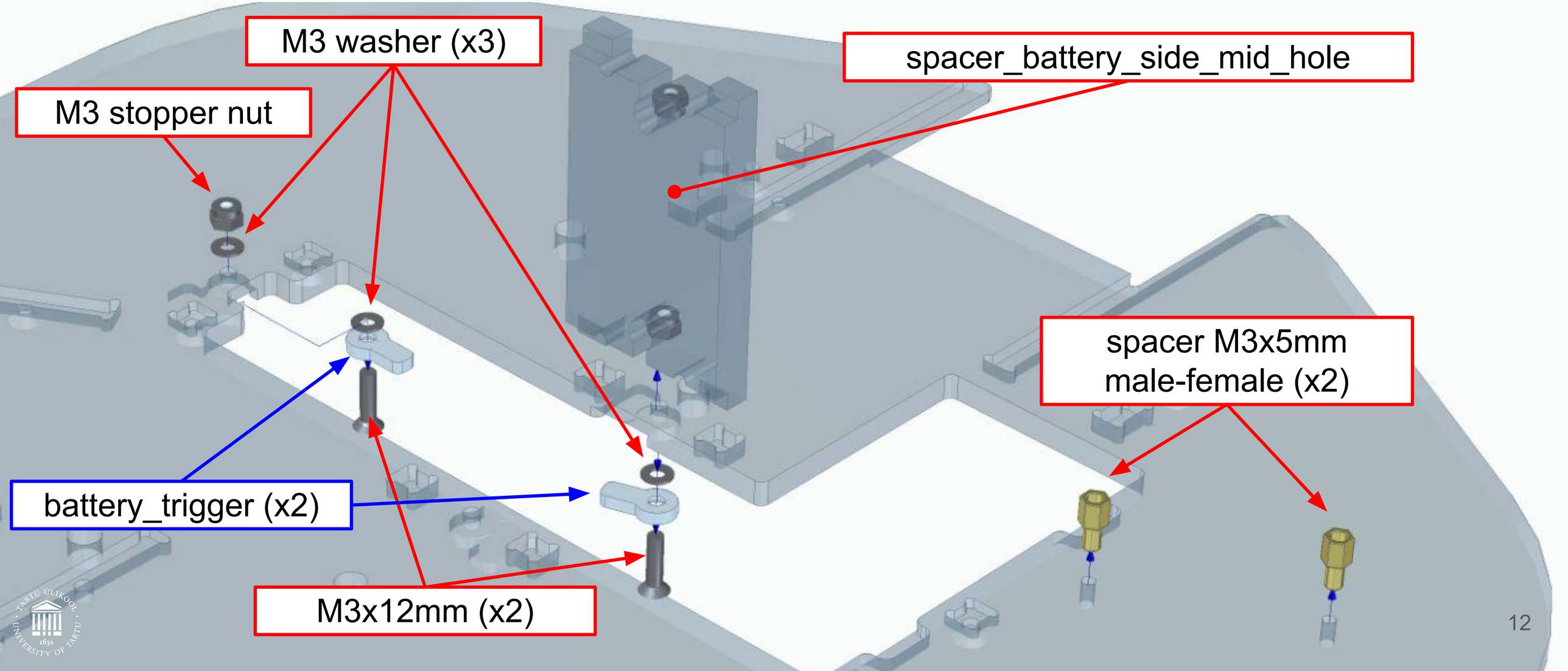

Mount electronics to the **spacer_battery_side_power_board** and then add this assembly to the **bottom_plate**

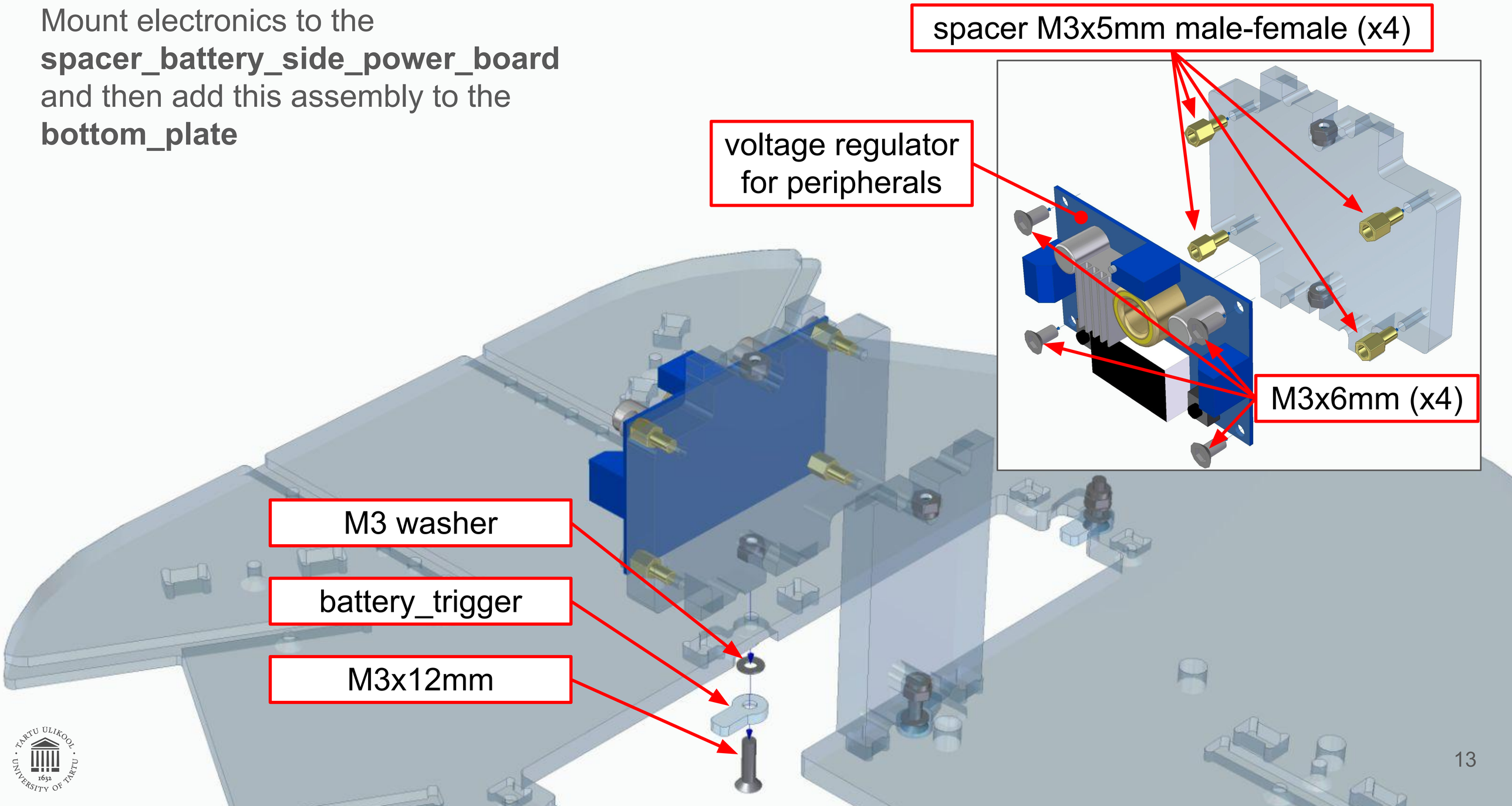

Use **M3x10mm (x4)** bolts to attach **spacer_battery_side_leg_hole (x2)**,
M3x12mm (x4) bolts to attach **spacer_bottom_top (x4)**,
M3x6mm (x2) bolts to attach the **power management board**.

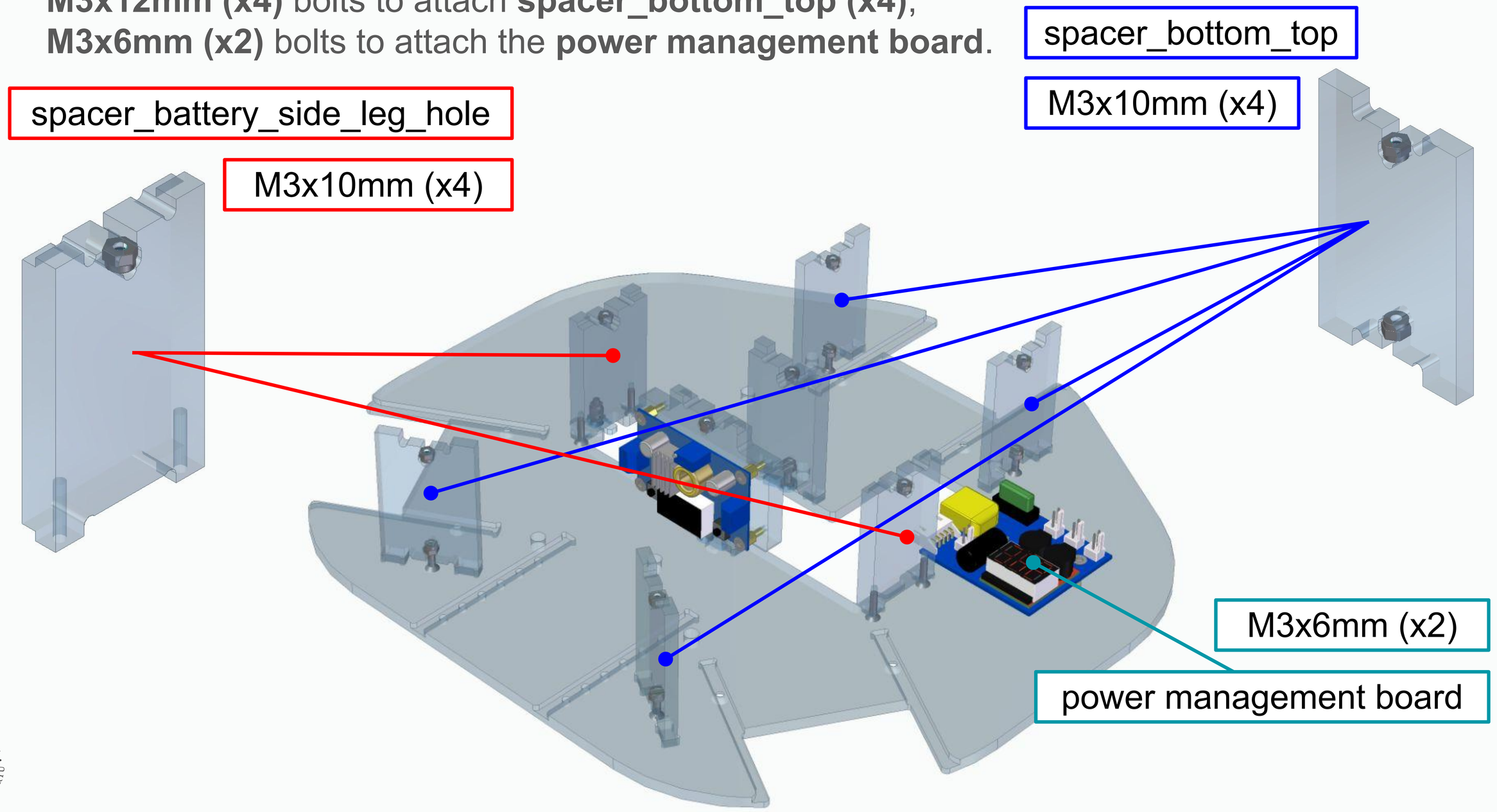

Mount the **STM32 NUCLEO-L476RG development board** on the **connector shield for NUCLEO-L476RG**.
Add the assembly to the **top_plate** with **M3x6mm (x2)** bolts**.**
Add the **E-stop** and **power** buttons to the **top_plate.**

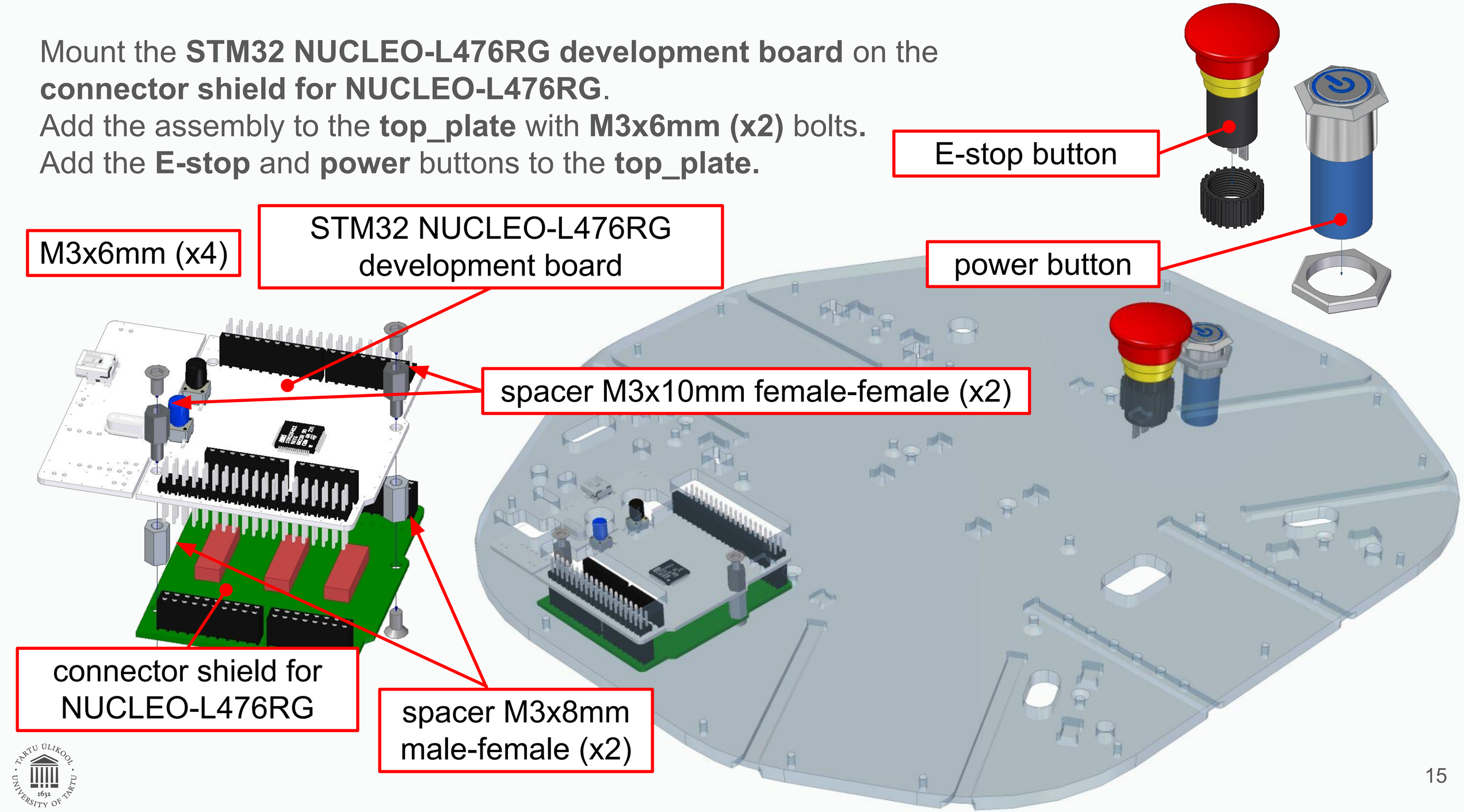

Connect and guide the cabling according to their function.
For motors, adhere to the numbering in red.
Attach the **top_plate** using **M3x12mm (x8)** bolts marked with red circles.

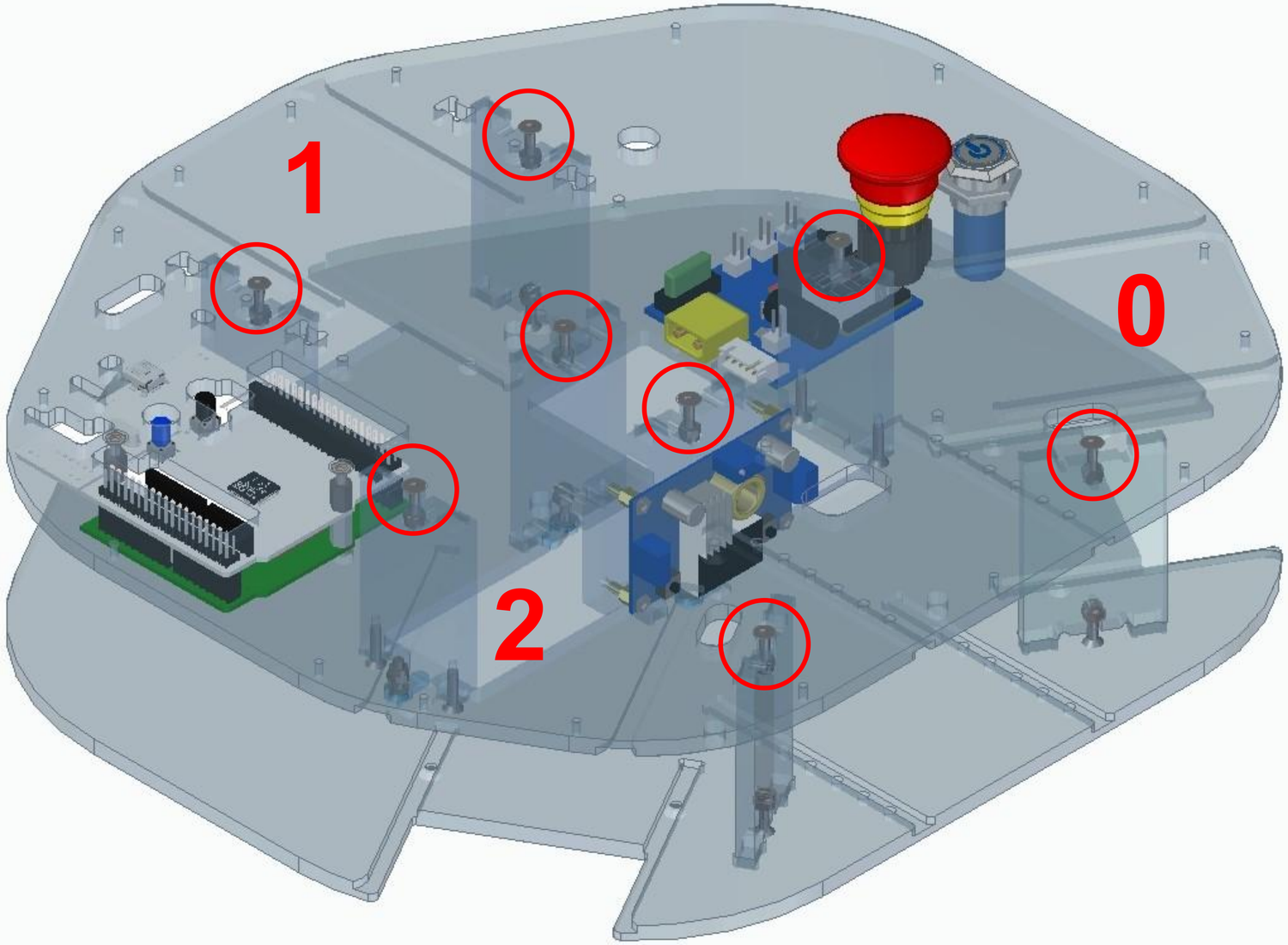

VEX Robotics omni wheel

Drill the hole in the center to 8mm

Use press tool or vises to add a shaft coupling

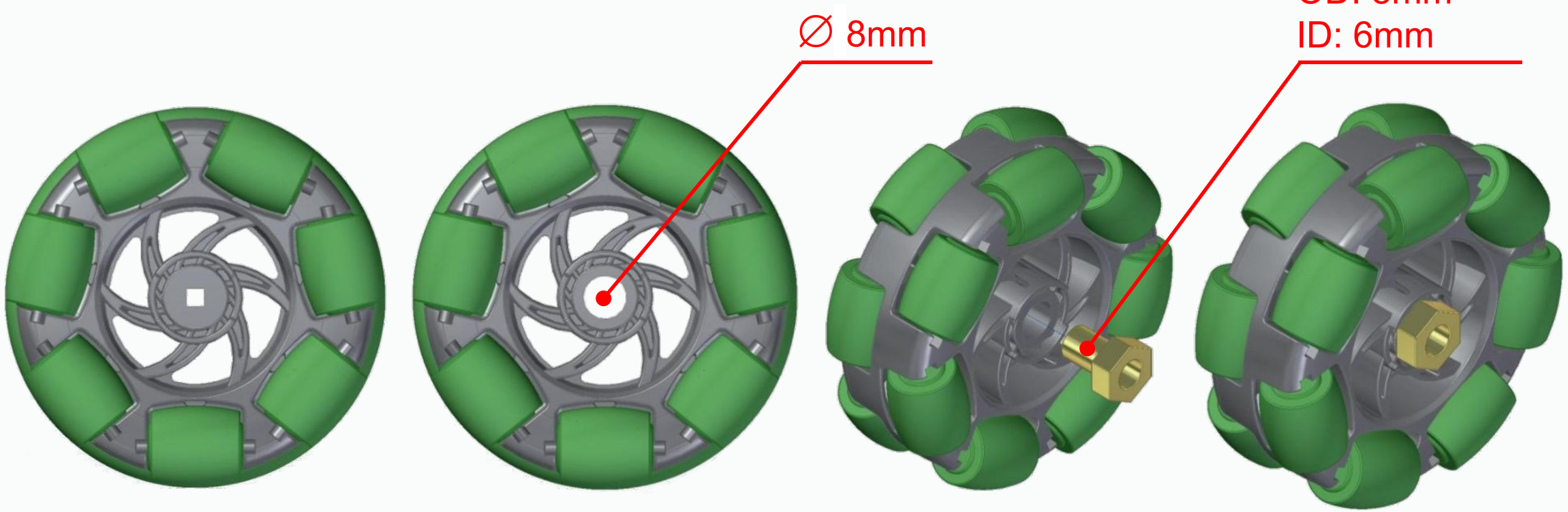

Assembly of wheel modules (x3)

(quantities are listed for assemblying a single module)

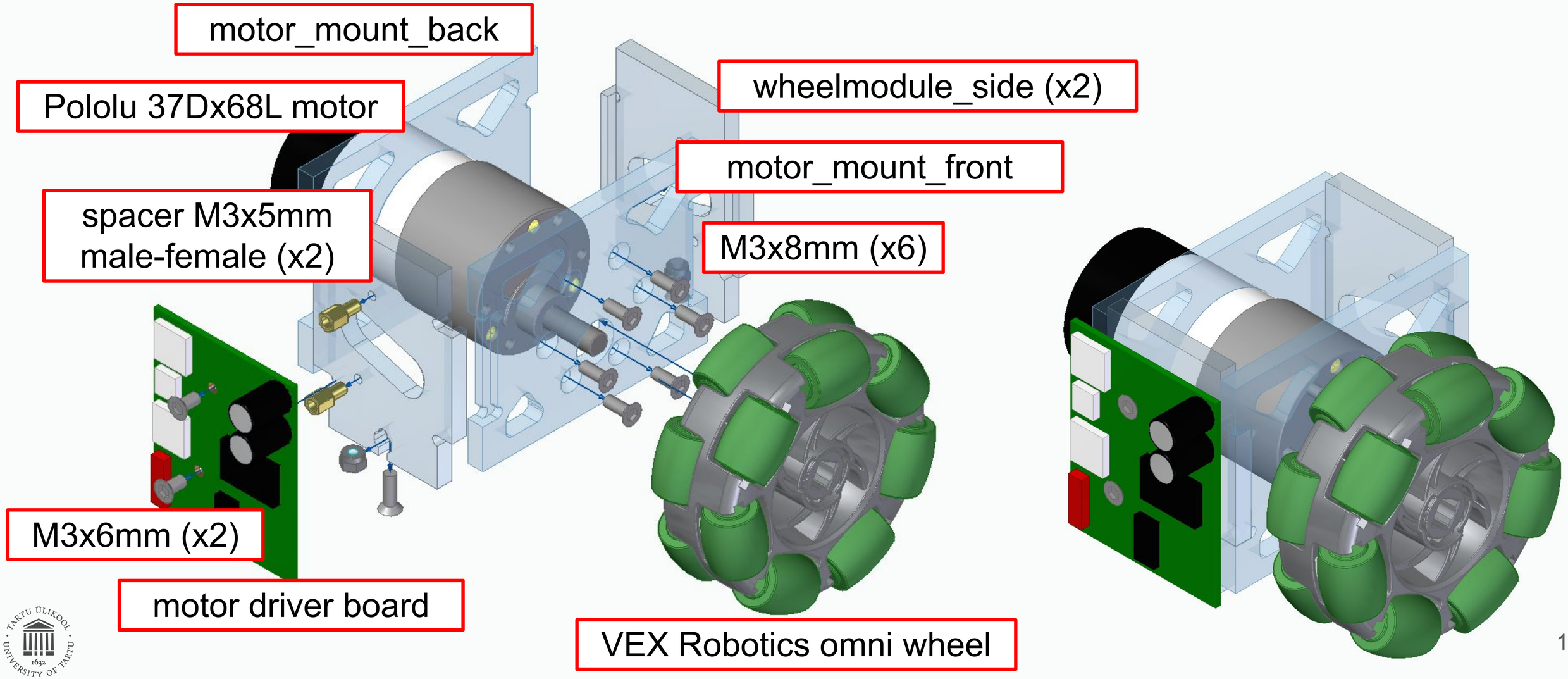

Use **M3x10mm (x6)** bolts to attach the wheel modules to the **bottom_plate**
Connect cabling according to the numbers in red

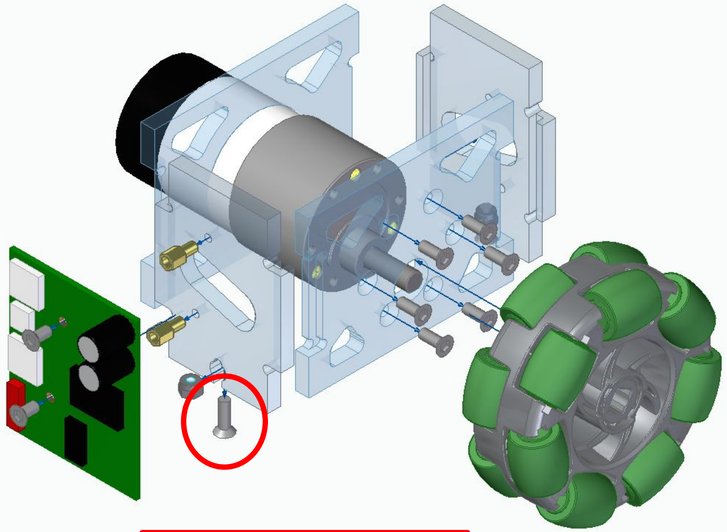

M3x10mm

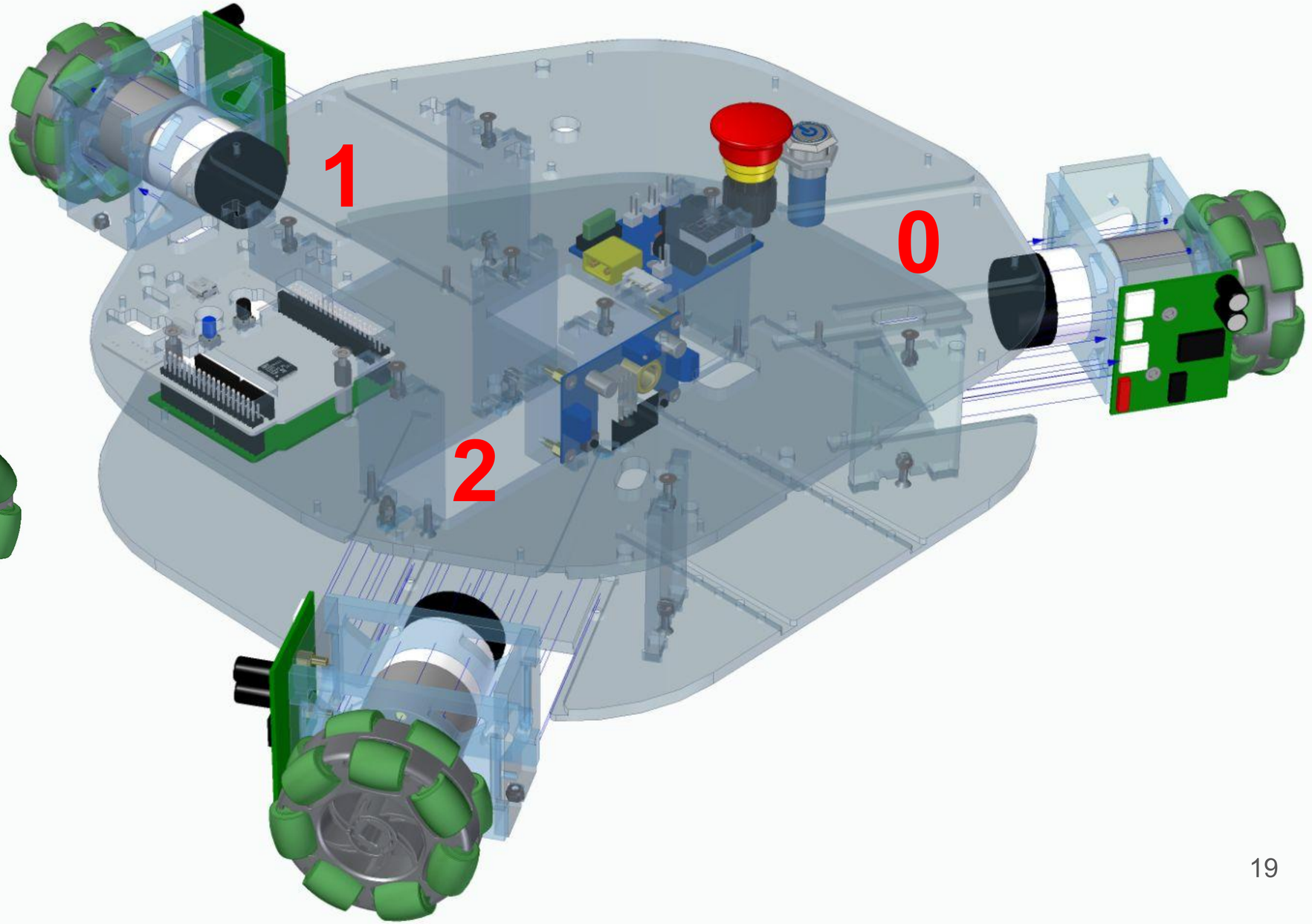

Use **M3x12mm (x2)** bolts and holes in the **bottom_plate** to mount the onboard computer

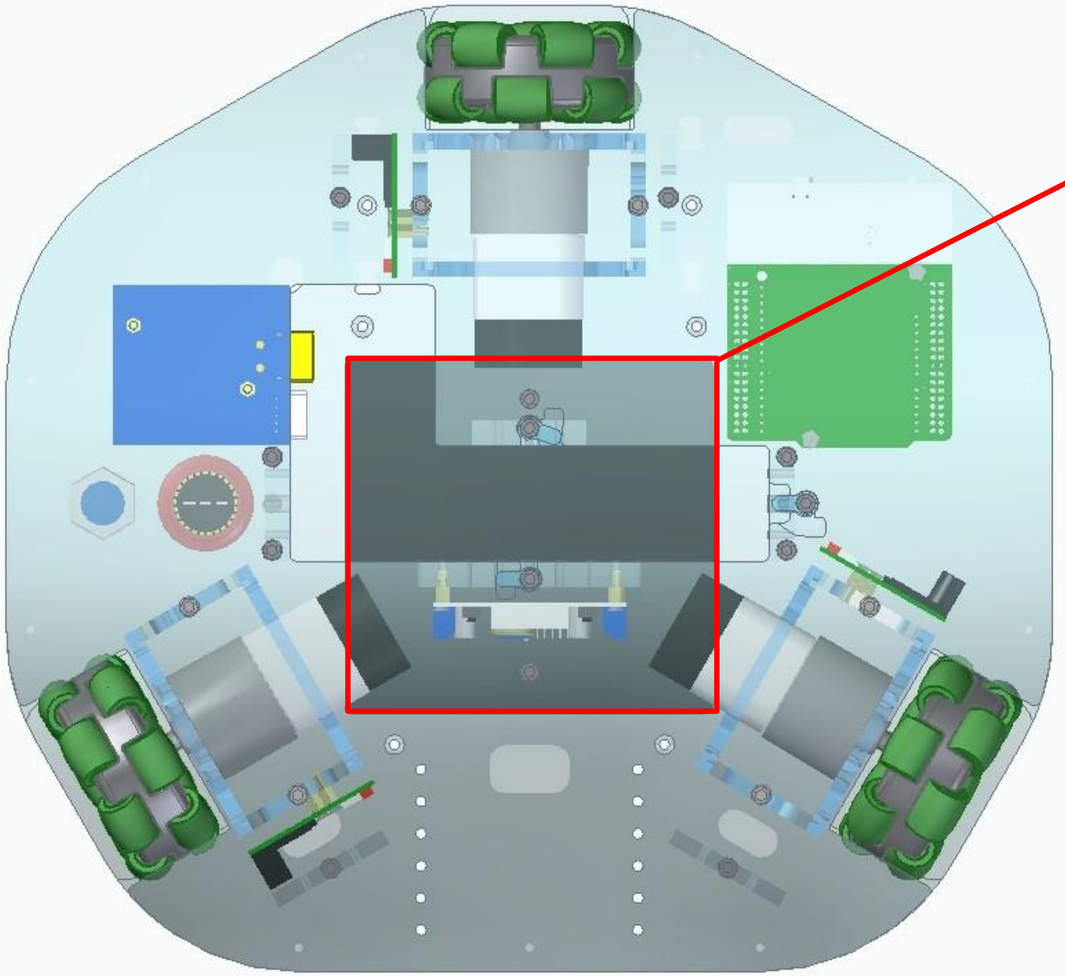

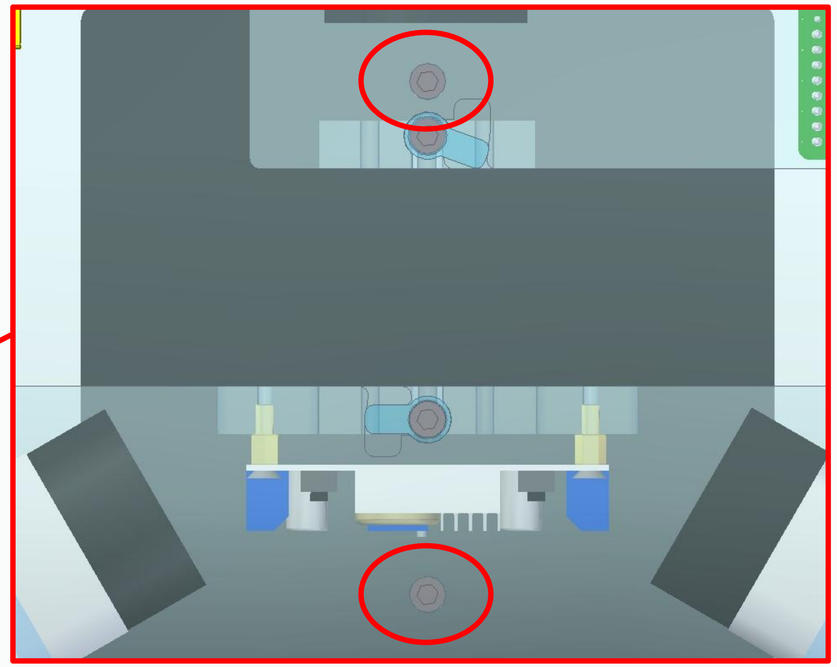

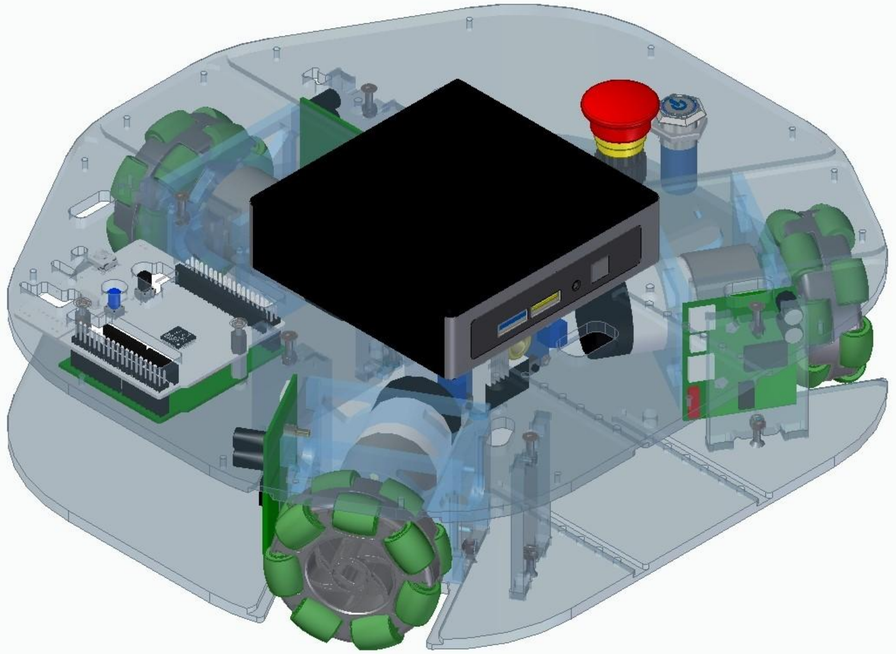

camera_tip_10x10mm (x2)

M3x6mm (x2)

M3 hat nut (x4)

cam_rear_panel

RealSense D435

cam_side_attach_left

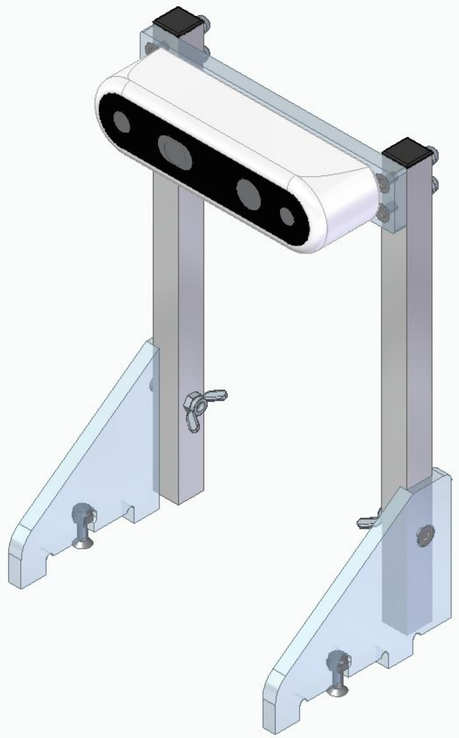

M3x16mm (x4)

camera_fixing_pipe_10x10mm (x2)

M4 butterfly nut (x2)

cam_side_attach_right

M4x16mm (x2)

M3 self locking nut (x2)

M3x12mm (x2)

Use **M3x12mm (x2)** bolts and holes in the **bottom_plate** to mount the **camera module**

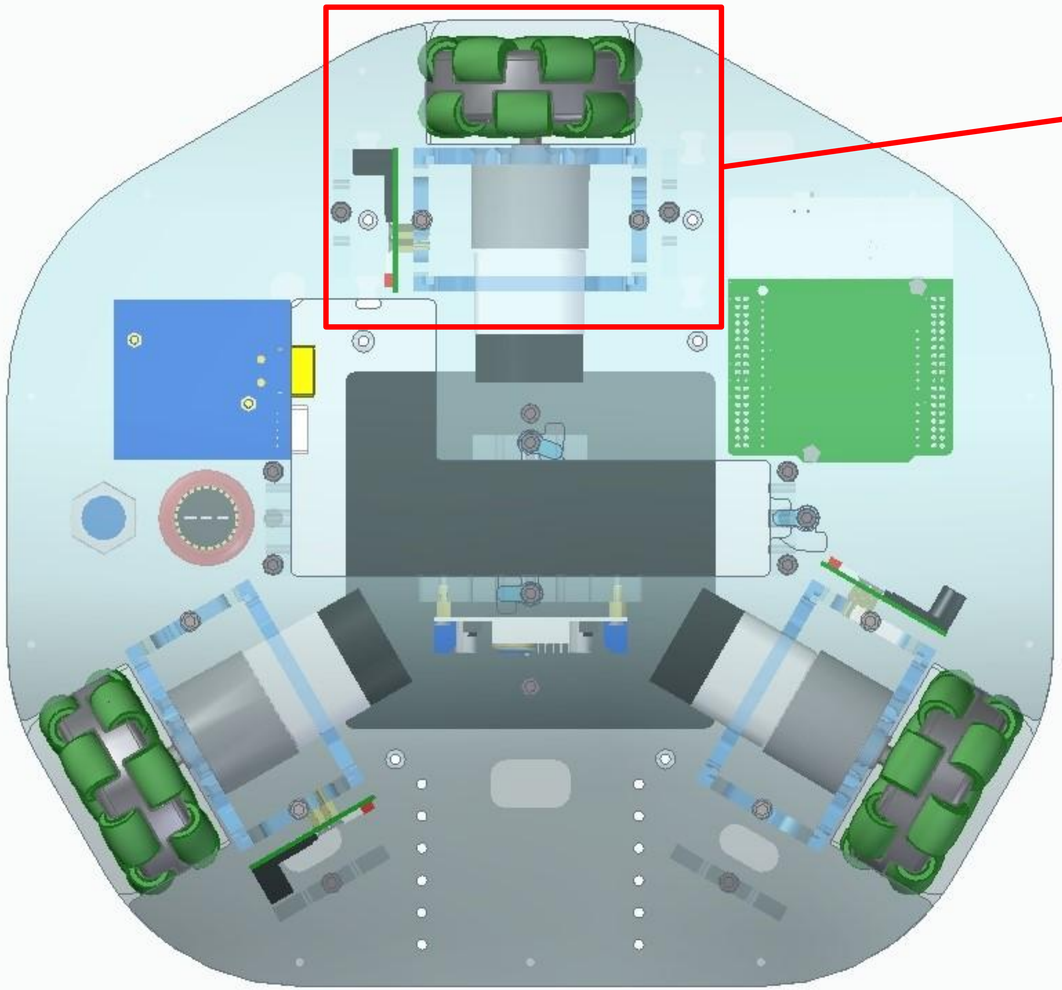

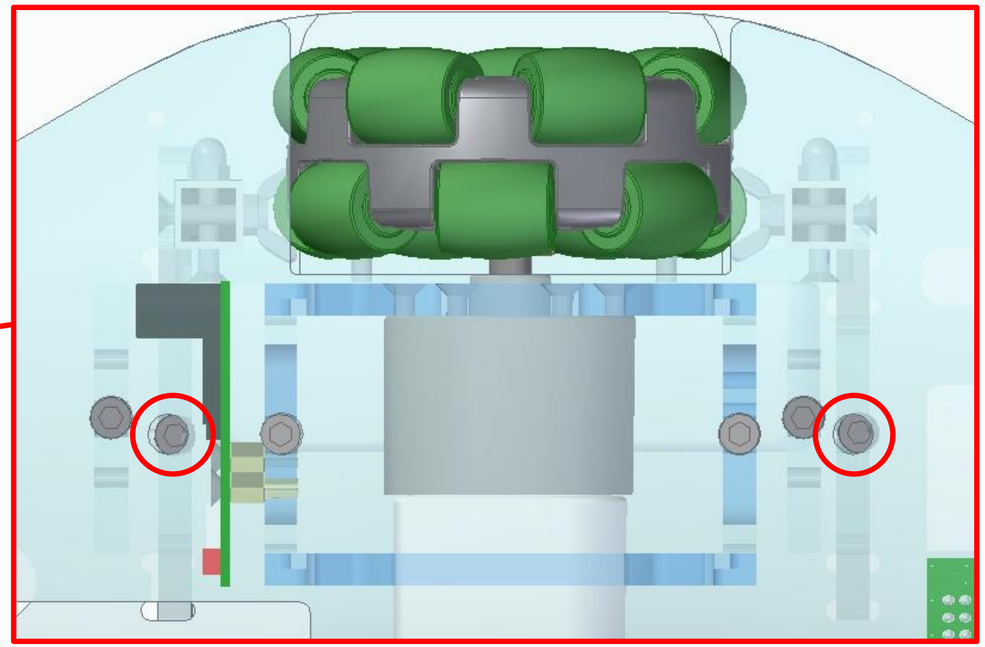

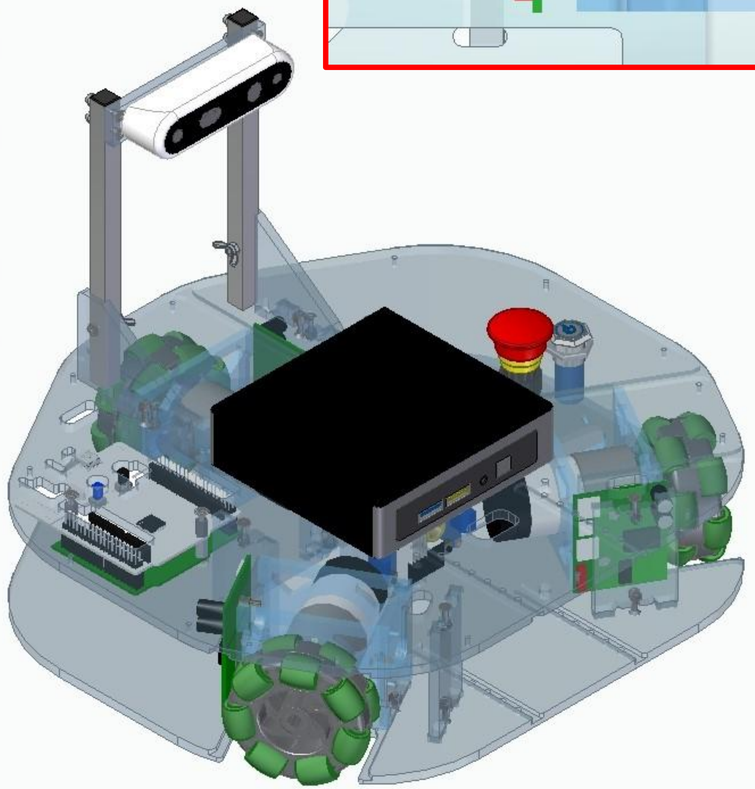

Mounting the **payload_tray**

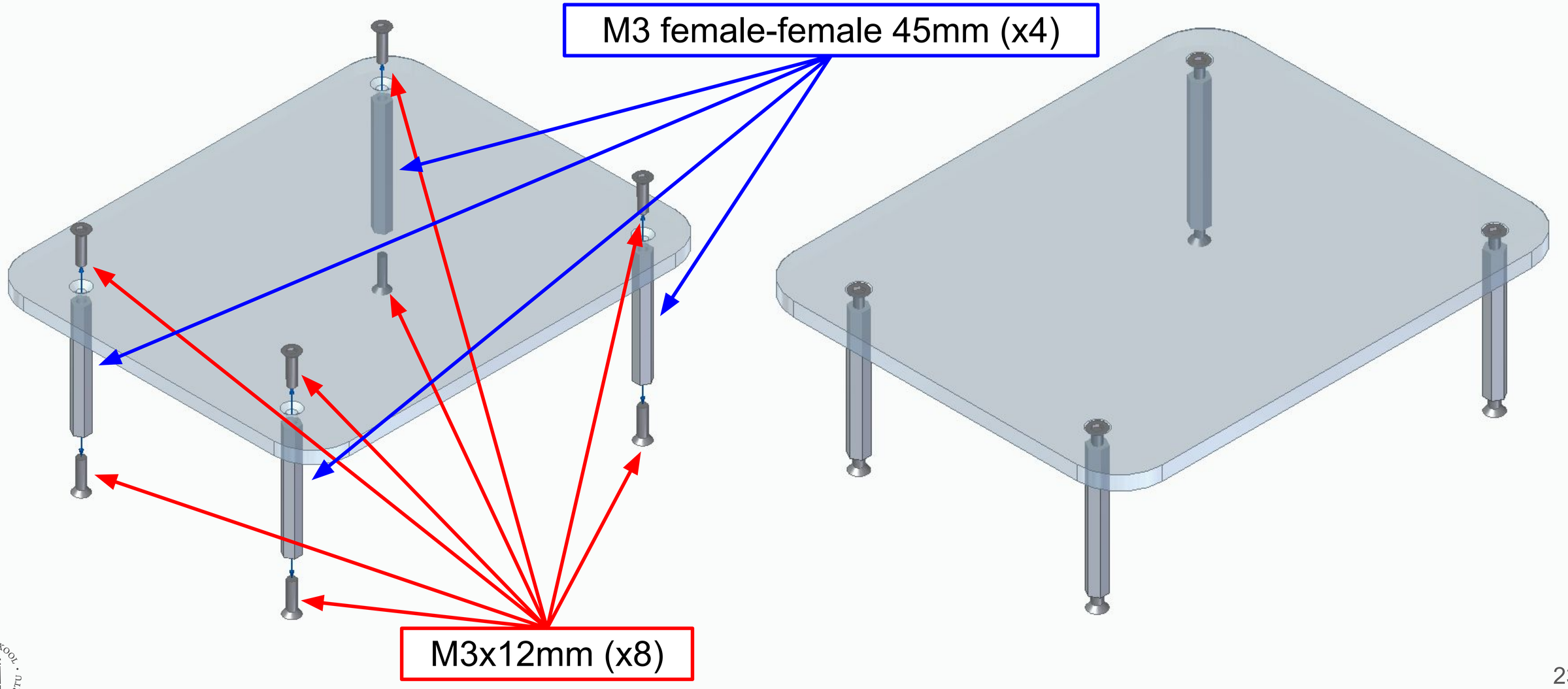

Use **M3x12mm (x4)** bolts and holes in the **bottom_plate** to mount the **payload_tray**

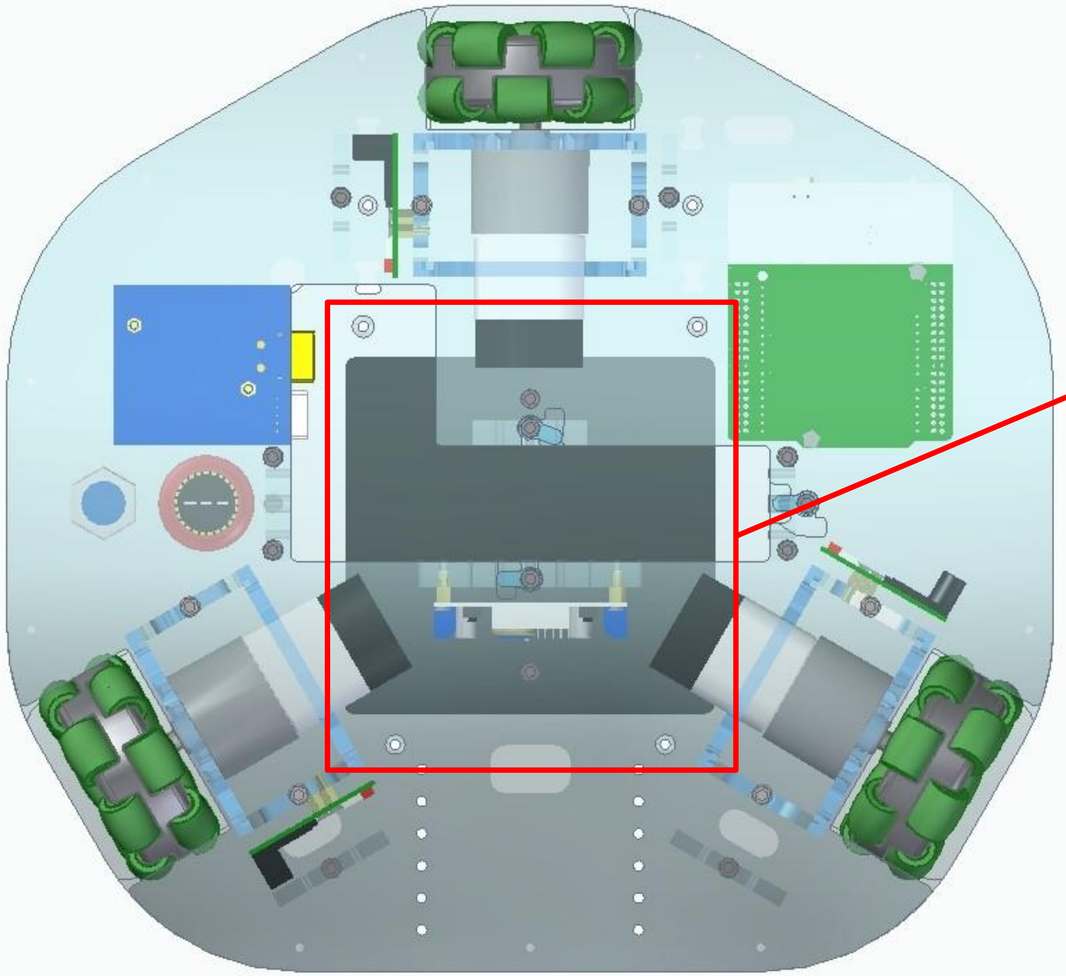

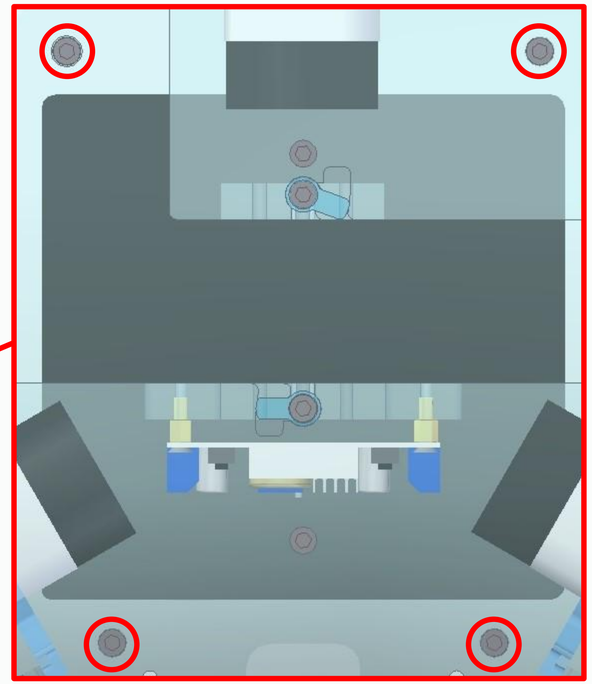

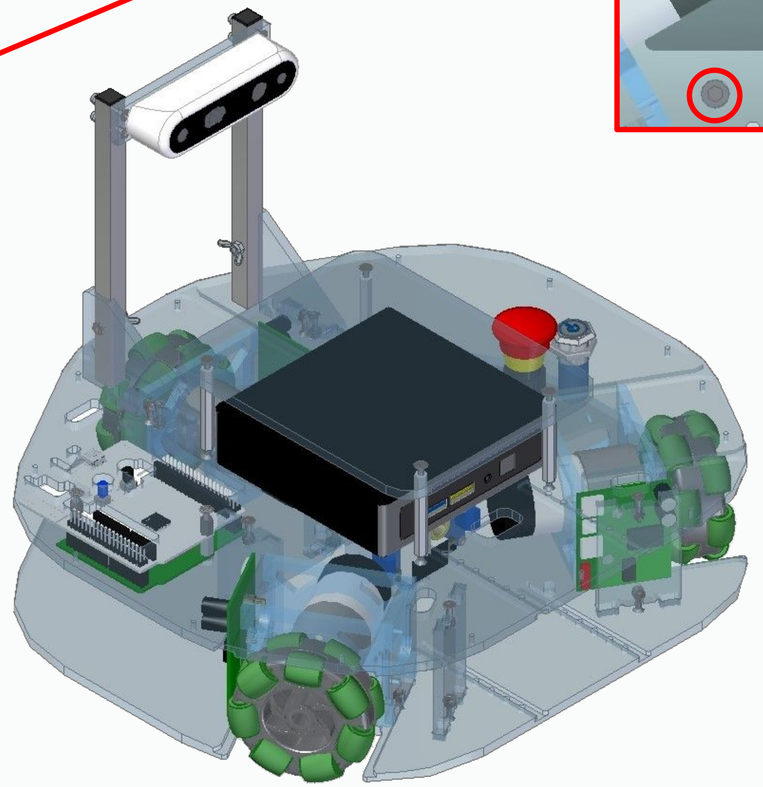

Battery installation: inserting the battery

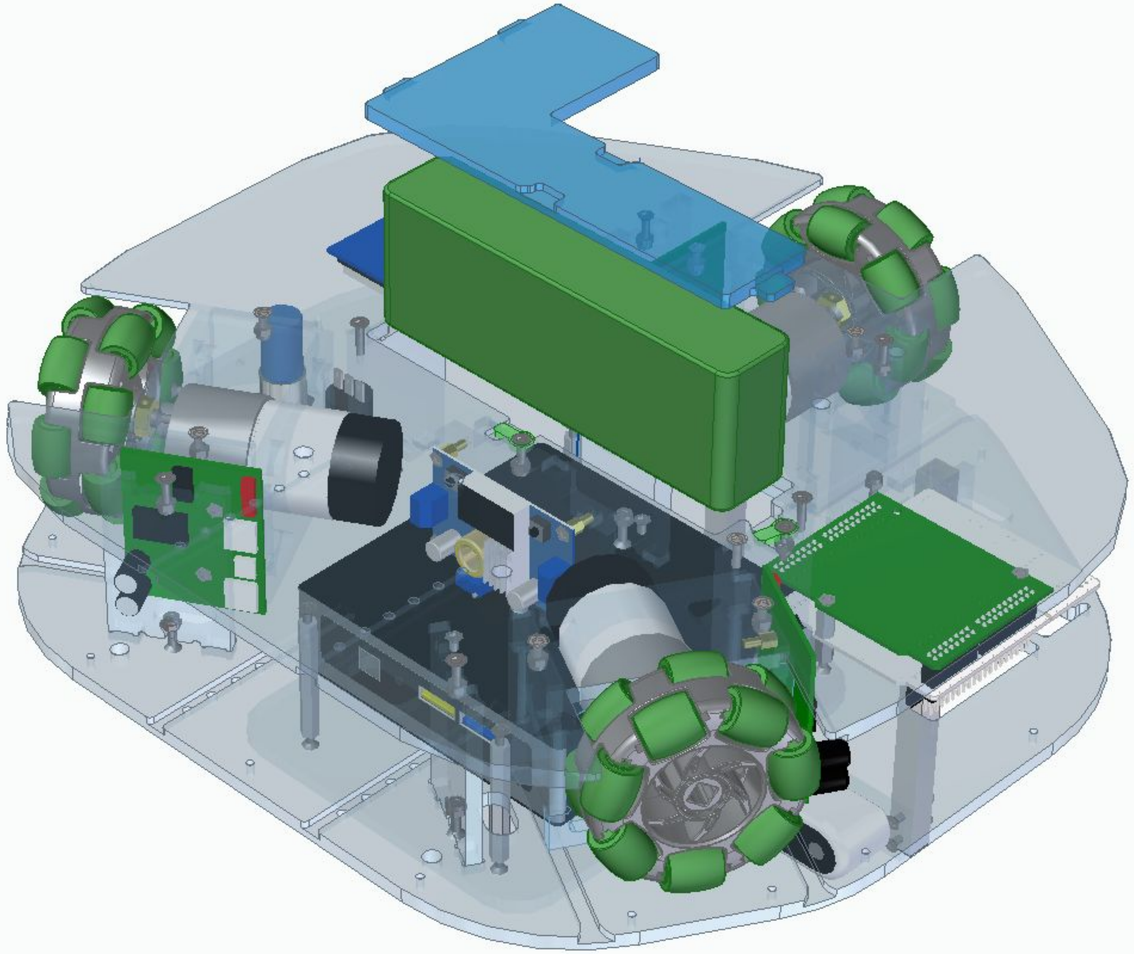

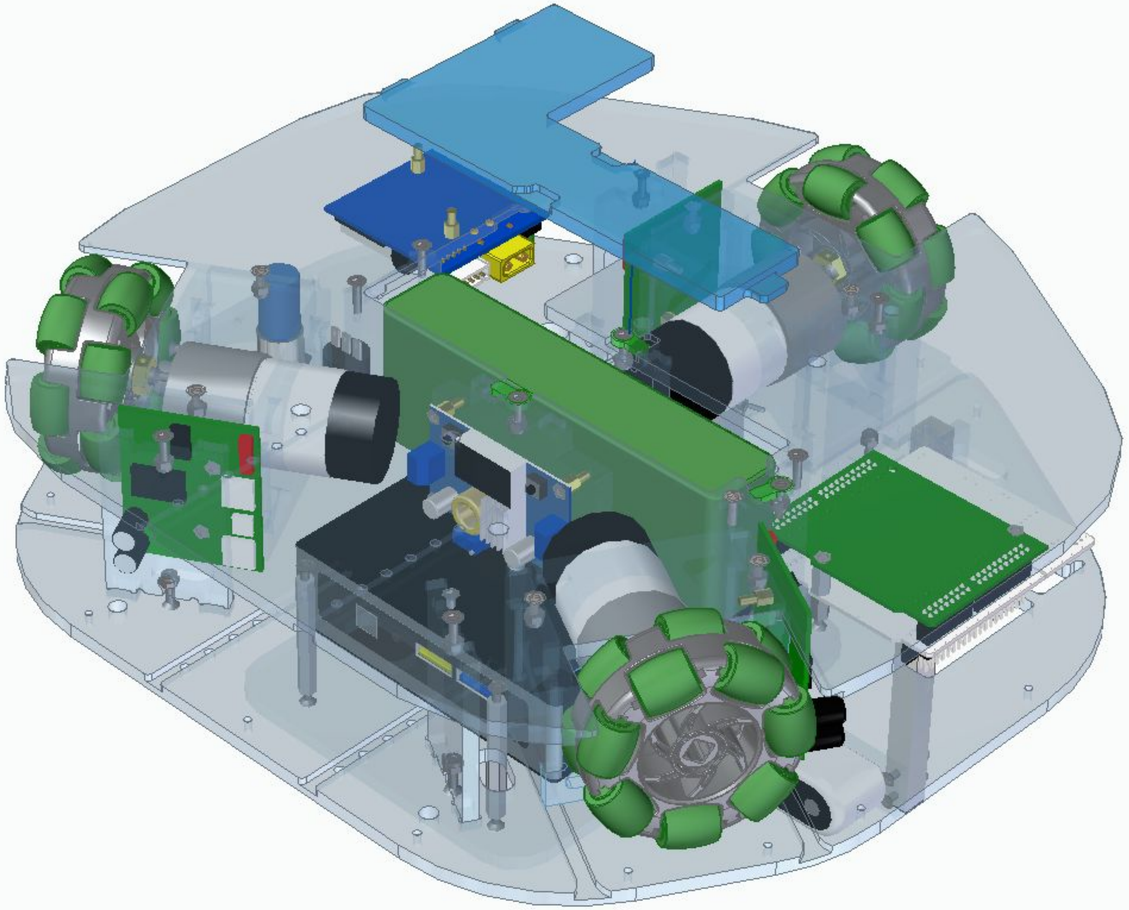

Battery installation: locking the lid

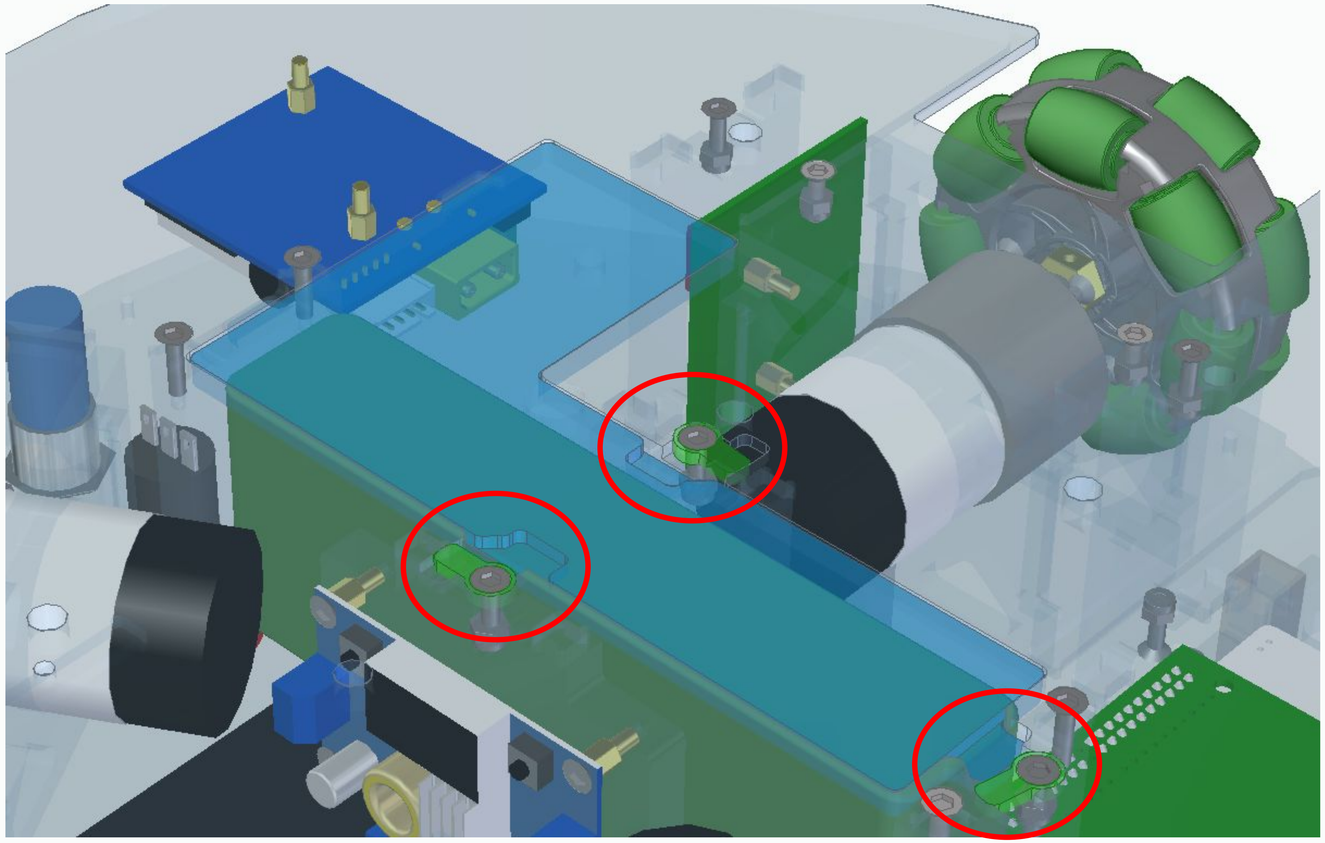

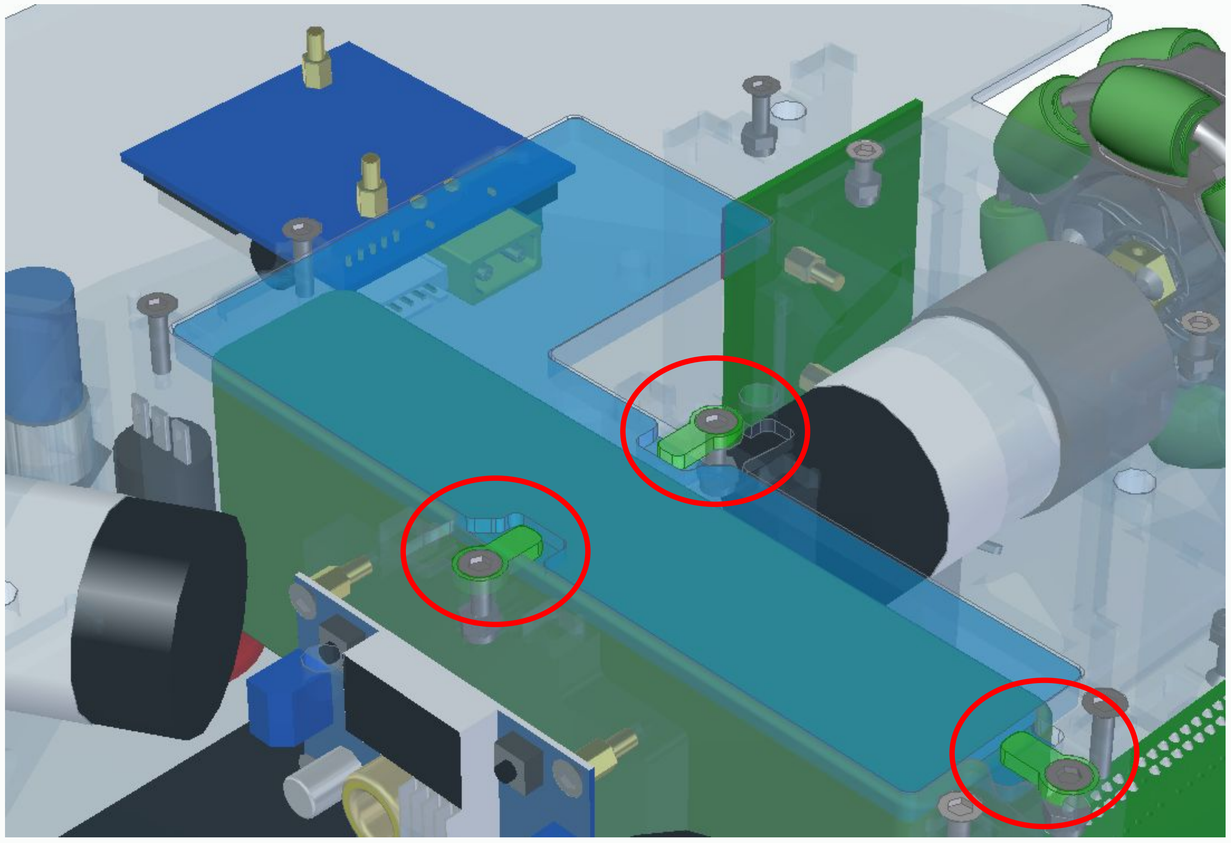

Full assembly

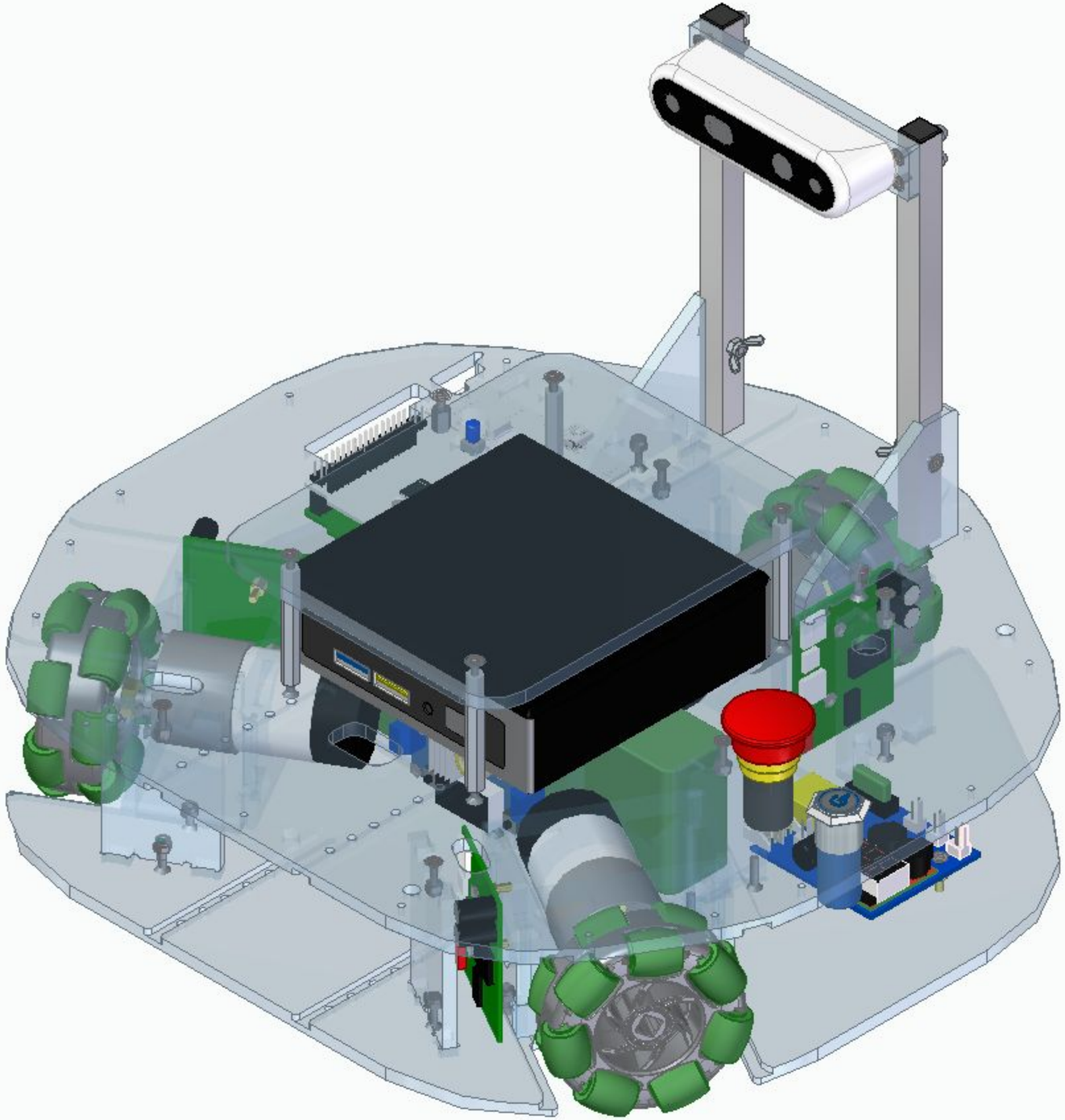

Latest information about ROBOTONT on GitHub

github.com/robotont

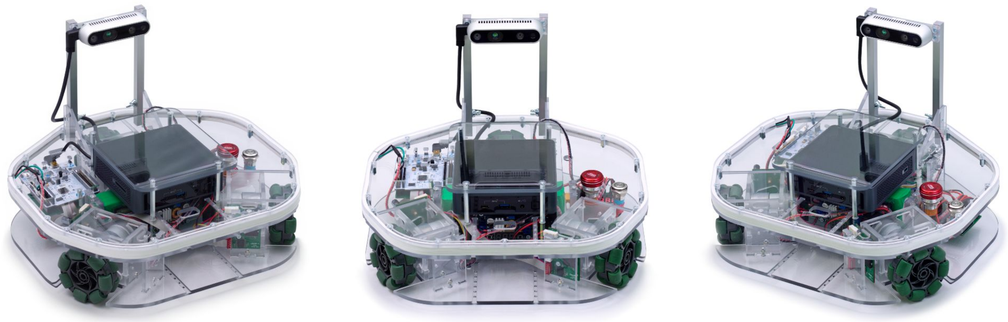

Supplement: Supplementary data 2 [file mmc2.pdf]
